# Variants in *CALD1*, *ESRP1*, and *RBFOX1* are associated with orofacial cleft risk

**DOI:** 10.1371/journal.pgen.1011581

**Published:** 2025-09-30

**Authors:** Jenna C. Carlson, Xinyi Zhang, Zeynep Erdogan-Yildirim, Terri H. Beaty, Azeez Butali, Carmen J. Buxó, Lord J.J. Gowans, Jacqueline T. Hecht, Ross E. Long, Lina Moreno, Jeffrey C. Murray, Ieda M. Orioli, Carmencita Padilla, George L. Wehby, Eleanor Feingold, Elizabeth J. Leslie-Clarkson, Seth M. Weinberg, Mary L. Marazita, John R. Shaffer

**Affiliations:** 1 Department of Human Genetics, School of Public Health, University of Pittsburgh, Pittsburgh, Pennsylvania, United States of America; 2 Department of Biostatistics and Health Data Science, School of Public Health, University of Pittsburgh, Pittsburgh, Pennsylvania, United States of America; 3 Department of Oral and Craniofacial Sciences, Center for Craniofacial and Dental Genetics, School of Dental Medicine, University of Pittsburgh, Pittsburgh, Pennsylvania, United States of America; 4 Department of Epidemiology, Johns Hopkins University, Baltimore, Maryland, United States of America; 5 Department of Oral Biology, Radiology, and Medicine, University of Iowa, Iowa City, Iowa, United States of America; 6 Dental and Craniofacial Genomics Core, School of Dental Medicine, University of Puerto Rico, San Juan, Puerto Rico; 7 Department of Biochemistry and Biotechnology, Kwame Nkrumah University of Science and Technology, Kumasi, Ghana; 8 Department of Pediatrics, McGovern Medical School University of Texas Health at Houston, Houston, Texas, United States of America; 9 Lancaster Cleft Palate Clinic, Lancaster, Pennsylvania, United States of America; 10 Department of Orthodontics & The Iowa Institute for Oral Health Research, University of Iowa, Iowa City, Iowa, United States of America; 11 Department of Pediatrics, University of Iowa, Iowa City, Iowa, United States of America; 12 INAGEMP (National Institute of Population Medical Genetics), Porto Alegre, Brazil; 13 ECLAMC (Latin American Collaborative Study of Congenital Malformations) at Department of Genetics, Federal University of Rio de Janeiro, Rio de Janeiro, Brazil; 14 Department of Pediatrics, College of Medicine, Institute of Human Genetics, National Institutes of Health, University of the Philippines Manila, Manila, The Philippines; 15 Philippine Genome Center, University of the Philippines System, Quezon, The Philippines; 16 Department of Health Management and Policy, College of Public Health, University of Iowa, Iowa, Iowa, United States of America; 17 Department of Statistics, Oregon State University, Corvallis, Oregon, United States of America; 18 Department of Human Genetics, Emory University School of Medicine, Atlanta, Georgia, United States of America; University of North Carolina at Chapel Hill, UNITED STATES OF AMERICA

## Abstract

Nonsyndromic orofacial clefts (OFCs) are common, heritable birth defects caused by both genetic and environmental risk factors. Despite the identification of many genetic loci harboring OFC-risk variants, there are many unknown genetic determinants of OFC. Furthermore, while the process of embryonic facial development is well characterized, the molecular mechanisms that underly it are not. This represents a major hurdle in understanding how disruptions in these biological processes result in OFC. Thus, we sought to identify novel OFC-risk loci through a genome-wide multi-ancestry study of five nested OFC phenotypes (isolated cleft lip [CLO], isolated cleft palate [CPO], cleft lip and palate [CLP], cleft lip with/without cleft palate [CL/P], and any cleft [ANY]) representing distinct cleft subtypes to identify subtype-specific signals and grouped types to maximize power to detect shared genetic effects. We performed genome-wide meta-analyses of these five OFC phenotypes from three cohorts totaling >14,000 individuals using METAL. In addition to replicating 13 known OFC-risk loci, we observed novel association in three regions: the 1p36.32 locus (lead variant rs584402, an intergenic variant, p_CLO_ = 3.14e-8), the 7q33 locus (lead variant rs17168118, an intronic variant in *CALD1*, p_CLP_ = 9.17e-9), and the 16p13.3 locus (lead variant rs77075754, an intronic variant in *RBFOX1*, p_CL/P _= 1.53e-9, p_ANY_ = 1.93e-9). We also observed a novel association within the known risk locus 8q22.1 that was independent of the previously reported signal (lead variant rs4735314, an intronic variant in *ESRP1*, p_CLP_ = 1.07e-9, p_CL/P_ = 3.88e-8). Next, we performed multi-tissue TWAS with s-MulTiXcan and identified four overlapping genes with significant genetically predicted transcription associated with OFC risk. These genes also overlapped the genome-wide significant association signals from the meta-analysis, including *CALD1* and *ESRP1* and known OFC-risk genes *TANC2* and *NTN1*. Each of the newly reported loci has potential regulatory effects, including evidence of craniofacial enhancer activity, that offer new clues as to the molecule mechanisms underlying embryonic facial development.

## Author summary

Orofacial clefts, including cleft lip and cleft palate, are common birth defects that can be caused by both genetic and environmental factors. While many of these factors are known, there are still significant gaps in our understanding of how and why clefts arise. To help address this deficiency, we measured association between variants across the genome and clefting in cases and controls from diverse genetic ancestries. We identified three new candidate genes (*CALD1*, *ESRP1*, and *RBFOX1*) that may be involved in cleft risk and reaffirmed the role of 12 previously reported risk genes. We also found evidence that clefting was associated with predicted gene expression at *CALD1* and *ESRP1* and that the associated variants in these genes were located near regions known to be involved in the regulation of gene expression in craniofacial tissues during development.

## Introduction

Orofacial clefts (OFC) are common, heritable birth defects caused by both genetic and environmental risk factors [1]. OFCs have a heterogeneous presentation and can occur in isolation (isolated) or simultaneously with other birth defects (non-isolated) [1–4]. Isolated OFCs, which affect ~1/700 births worldwide, consist of clefts of the lip and/or palate. OFCs arise through disruption of the formation of the lip and/or palate, which involves a complex series of events spanning weeks 4–10 of human development [2]. While this process is well characterized, the underlying molecular mechanisms that lead to clefting are not. Knowledge of the various genes and pathways involved is critical to this endeavor.

The genetic etiology of OFCs is complex, characterized by both allelic and locus heterogeneity [5]. Moreover, the three main forms of nonsyndromic OFC (cleft lip only or CLO, cleft palate only or CPO, and cleft lip and palate or CLP) are believed to have both shared and subtype-specific genetic risk factors [1,3,4]. Despite the identification of over 60 genetic loci harboring OFC-risk variants [6], these only account for an estimated 25% of the heritability of isolated OFC [7], suggesting that there are more genetic determinants yet to be discovered. Moreover, the biological interpretation of these loci is challenging since most OFC-risk variants fall outside protein-coding gene regions, making their functional impact a challenge to decipher.

In addition to differences across cleft subtypes, there are large differences in OFC prevalence according to genetic ancestry [8,9]. For example, OFC risk is higher in individuals of recent East Asian ancestry compared to individuals of recent European or Sub-Saharan African ancestry. Such differences make it essential to include diverse cohorts in our genetic analyses, yet most studies of OFC inadequately address this concern.

In the current study, we attempt to address some of these gaps by conducting genome-wide meta-analyses of three ancestrally diverse cohorts of individuals and trios with isolated OFCs (hereafter simply referred to as OFCs).

## Results

We performed genome-wide meta-analyses from three studies totaling 14,481 individuals from diverse genetic ancestries for five cleft phenotypes: CLO, CPO, CLP, cleft lip with or without cleft palate (CL/P), and any cleft (ANY) (Fig A - E in S1 Text). We observed genome-wide significant (p < 5e-8) signals at 16 loci (Table 1), including 13 known OFC-risk loci: *PAX7* (1p36.3), *ARHGAP29* (1p22.1), *IRF6* (1q32.2), *THADA* (2p21), *FGF10* (5p12), 8q21.3, 8q22.1, 8q24.21, *VAX1* (10q25.3), *SPRY2* (13q31.1), *NTN1* (17p13.1), *TANC2* (17q23.2), and *MAFB* (20q12). At some of these loci, there were multiple independent signals, including two at *PAX7*, five at *IRF6*, five at 8q24.21, three at *VAX1*, two at *SPRY2*, and two at *NTN1* (17p13.1) (Table 1).

**Table 1:** Results from genome-wide all ancestry meta-analysis for each independent lead variant demonstrating genome-wide significance (p < 5e-8) in at least one phenotype. Columns include Chromosome (Chr), position (hg38), reference and alternate (effect) alleles (Ref/Alt), rsID, candidate gene (either the gene nearest the variant or a previously implicated risk gene), direction of effect (Dir) on any cleft for POFC1, POFC2, and GENEVA OFC, respectively (? indicates variant was not present in that study), and p-values and from meta-analysis across each phenotype definition.

Region	Chr	Pos	Ref	Alt	rsID	Candidate Gene	Dir	P ANY	P CL/P	P CLP	P CLO	P CPO
**1p36.32**	**1**	**5238225**	**C**	**T**	**rs584402**	**AJAP1**	**++-**	**4.74E-03**	**1.02E-03**	**7.09E-02**	**3.14E-08**	**4.81E-01**
1p36.13	1	18645140	G	C	rs56675509	PAX7	+++	6.84E-10	1.41E-12	2.08E-09	1.67E-07	8.53E-01
1p36.13	1	18646288	TTC	T	rs10546636	PAX7	+++	1.11E-10	1.11E-13	4.43E-11	2.18E-07	3.47E-01
1p22.1	1	94092554	G	T	rs66515264	ARHGAP29	++?	6.46E-09	3.30E-10	2.82E-08	1.94E-05	2.11E-01
1q32.2	1	209846668	C	A	rs3766612	IRF6	+++	5.50E-10	9.61E-12	1.71E-05	2.19E-12	6.07E-01
1q32.2	1	209942373	C	A	rs201670404	IRF6	---	2.00E-08	7.63E-11	2.56E-09	6.16E-05	4.22E-01
1q32.2	1	209798853	G	A	rs4844494	IRF6	---	3.95E-18	1.00E-22	1.74E-16	3.13E-12	1.29E-01
1q32.2	1	209815644	G	A	rs17015268	IRF6	---	4.30E-18	5.42E-23	1.31E-16	1.05E-12	8.93E-02
1q32.2	1	209815936	G	A	rs77542756	IRF6	---	4.12E-18	7.23E-23	1.74E-16	9.96E-13	9.63E-02
2p21	2	43286897	G	A	rs11900952	THADA	---	3.24E-08	5.83E-09	1.44E-07	2.21E-05	3.35E-01
5p12	5	44263295	C	T	rs6883600	FGF10	++?	4.21E-07	4.23E-08	1.40E-05	3.12E-07	4.57E-01
**7q33**	**7**	**134892089**	**A**	**G**	**rs17168118**	**CALD1**	**++?**	**1.44E-06**	**5.77E-08**	**9.17E-09**	**2.81E-03**	**8.55E-01**
8q21.3	8	87892130	C	T	rs11786656	DCAF4L2	---	8.98E-09	1.94E-11	3.03E-09	6.68E-06	5.92E-01
**8q22.1**	**8**	**94656529**	**T**	**C**	**rs4735314**	**ESRP1**	**---**	**7.46E-06**	**3.88E-08**	**1.07E-09**	**1.53E-02**	**2.82E-01**
8q24.21	8	128952627	G	A	rs17242358	CCDC26	+++	2.37E-27	4.14E-35	9.74E-25	2.45E-19	4.41E-01
8q24.21	8	128754576	A	G	rs2349688	CCDC26	---	4.31E-08	2.68E-10	9.18E-08	2.23E-06	NA
8q24.21	8	128965218	T	C	rs1470206	CCDC26	---	1.34E-13	1.34E-15	2.16E-10	2.56E-08	9.10E-01
8q24.21	8	128886123	A	T	rs2119756	CCDC26	??-	9.49E-05	4.49E-08	1.91E-03	4.67E-07	6.37E-02
8q24.21	8	128969112	C	T	rs11997787	CCDC26	+++	1.03E-14	4.40E-18	3.06E-12	7.41E-09	4.46E-01
10q25.3	10	117076565	AC	A	rs5788208	VAX1	+++	1.42E-08	1.24E-09	5.33E-08	5.90E-05	8.73E-01
10q25.3	10	117081903	G	T	rs11197889	VAX1	+++	9.14E-09	9.15E-10	5.00E-08	4.73E-05	9.17E-01
10q25.3	10	117081904	C	T	rs11197890	VAX1	+++	7.67E-09	8.08E-10	5.11E-08	4.16E-05	9.29E-01
13q31.1	13	80126941	G	C	rs11842594	SPRY2	+++	2.50E-07	1.51E-09	1.14E-10	3.93E-02	5.57E-01
13q31.1	13	80127350	T	C	rs1854110	SPRY2	---	1.49E-07	1.38E-09	1.54E-10	3.53E-02	6.62E-01
**16p13.3**	**16**	**6429186**	**A**	**G**	**rs77075754**	**RBFOX1**	**??+**	**1.93E-09**	**1.53E-09**	**1.31E-06**	**2.36E-04**	**NA**
17p13.1	17	9026902	AACCCAAAACCCAC	A	rs11273201	NTN1	---	2.19E-12	4.22E-14	2.42E-13	3.71E-05	5.19E-01
17p13.1	17	9017282	T	C	rs4791330	NTN1	---	1.39E-08	4.72E-11	1.90E-12	1.25E-02	4.23E-01
17q23.2	17	62971963	C	T	rs72843143	TANC2	---	6.04E-05	3.69E-08	5.78E-06	5.33E-05	1.18E-01
20q12	20	40613502	G	C	rs11700188	MAFB	??-	1.98E-10	1.20E-11	4.56E-11	2.25E-02	2.76E-01

Abbreviations ANY: any cleft, CL/P: cleft lip with/without cleft palate, CLP: cleft lip and palate, CLO: cleft lip only, CPO: cleft palate only. Bold values indicate novel associations.

**Fig 1:**
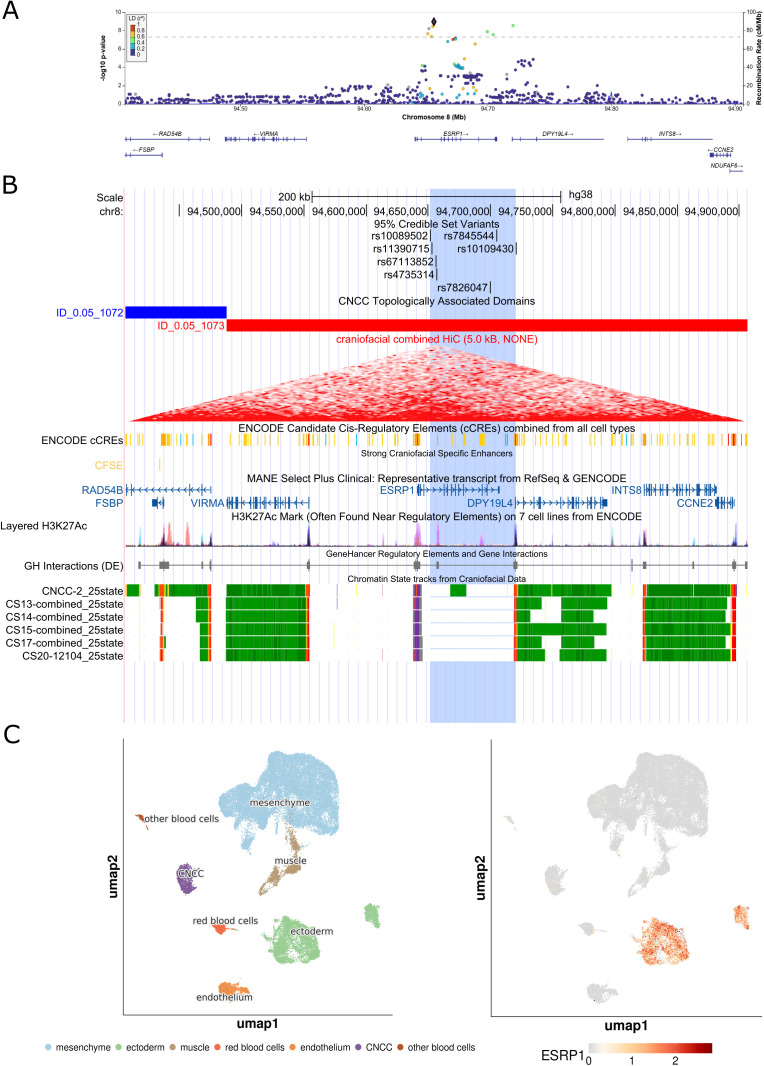
Integrated genomic analysis of the association at 8q22.1 with cleft lip and palate (CLP). (A) Regional association plots of meta-analyses for 8q22.1 with cleft lip and palate (CLP) generated using LocusZoom [11]. Association results were obtained via fixed effects p-value based meta-analysis using METAL [12]. Variants are colored based on LD with the top variant in each region (shown in purple) based on the 1000 Genomes Phase 3 all populations references. (B) UCSC Genome Browser view, annotated with 95% credible set variants, cranial neural crest cell (CNCC) specific topologically associated domains (TADs) derived from craniofacial Hi-C data [13], candidate cis-regulatory elements from non-craniofacial cell types (ENCODE) as well as strong craniofacial specific enhancers [13], gene-enhancer interactions (GeneHancer), and chromatin state tracks from craniofacial data [14]. (C) results from single-cell RNA sequencing of craniofacial tissue [15] including cell information (left) and gene expression levels for *ESRP1* (right).

**Fig 2:**
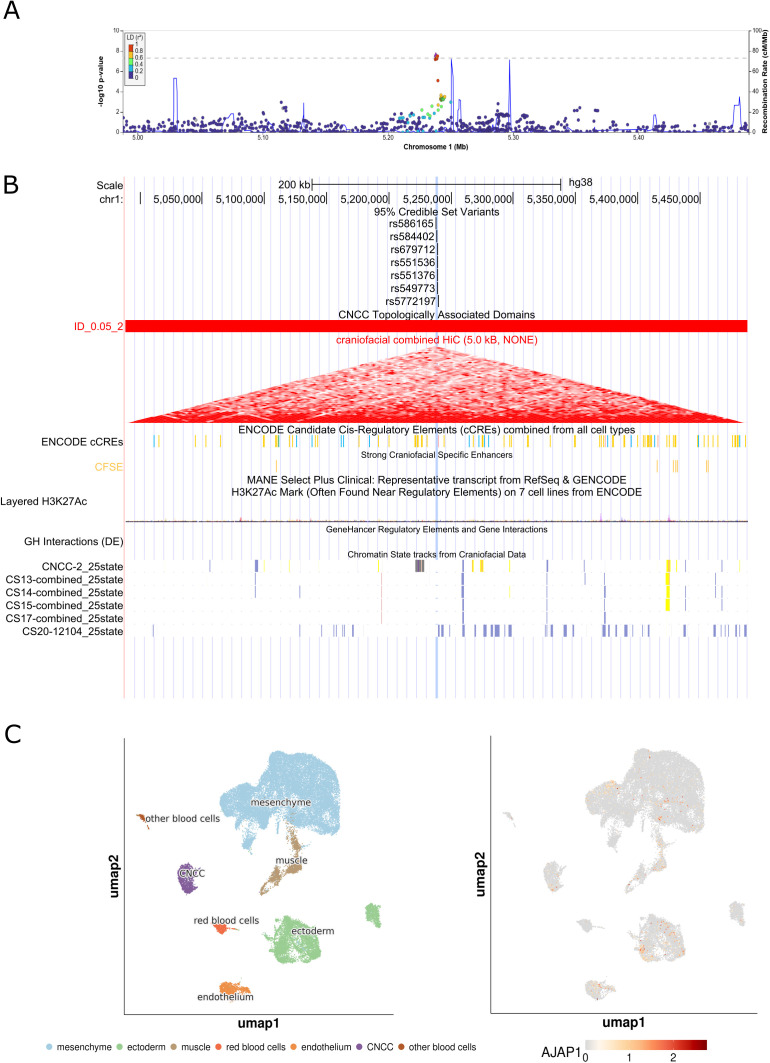
Integrated genomic analysis of the association at 1p36.32 with cleft lip (CLO). (A) Regional association plots of meta-analyses for 1p36.32 with cleft lip (CLO) generated using LocusZoom [11]. Association results were obtained via fixed effects p-value based meta-analysis using METAL [12]. Variants are colored based on LD with the top variant in each region (shown in purple) based on the 1000 Genomes Phase 3 all populations references. (B) UCSC Genome Browser view, annotated with 95% credible set variants, cranial neural crest cell (CNCC) specific topologically associated domains (TADs) derived from craniofacial Hi-C data [13], candidate cis-regulatory elements from non-craniofacial cell types (ENCODE) as well as strong craniofacial specific enhancers [13], gene-enhancer interactions (GeneHancer), and chromatin state tracks from craniofacial data [14]. (C) results from single-cell RNA sequencing of craniofacial tissue [15] including cell information (left) and gene expression levels for *AJAP1* (right).

**Fig 3:**
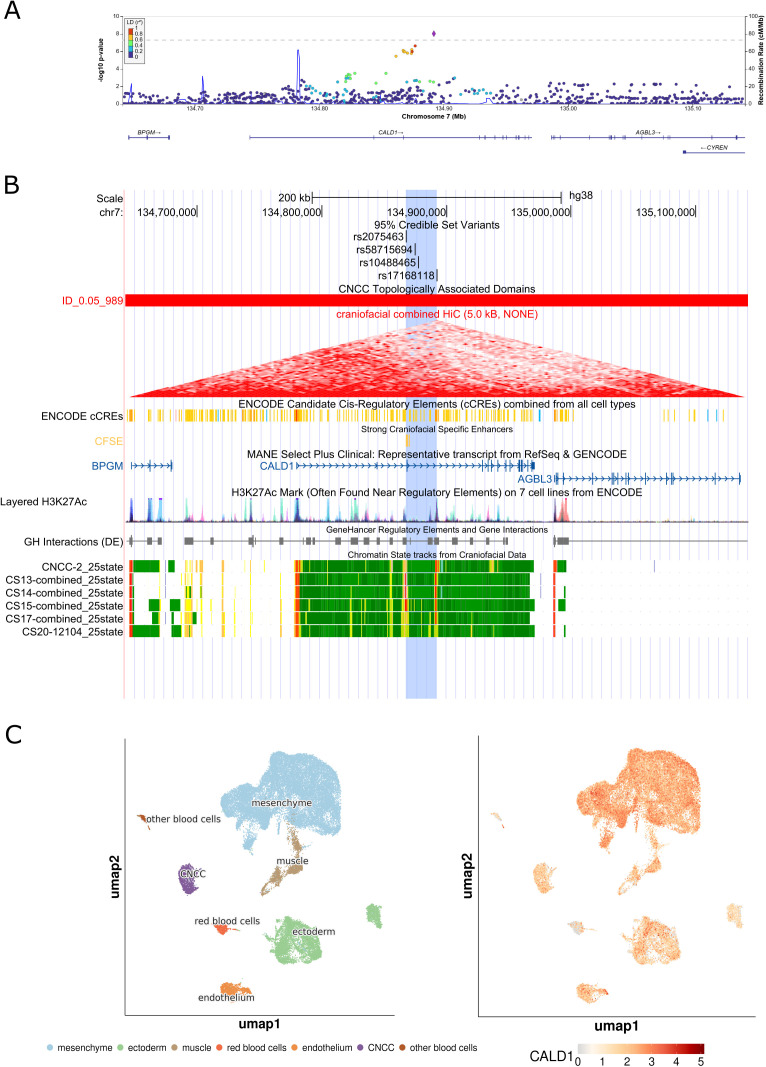
Integrated genomic analysis of the association at 7q33 with cleft lip and palate (CLP). (A) Regional association plots of meta-analyses for 7q33 with cleft lip and palate (CLP) generated using LocusZoom [11]. Association results were obtained via fixed effects p-value based meta-analysis using METAL [12]. Variants are colored based on LD with the top variant in each region (shown in purple) based on the 1000 Genomes Phase 3 all populations references. (B) UCSC Genome Browser view, annotated with 95% credible set variants, cranial neural crest cell (CNCC) specific topologically associated domains (TADs) derived from craniofacial Hi-C data [13], candidate cis-regulatory elements from non-craniofacial cell types (ENCODE) as well as strong craniofacial specific enhancers [13], gene-enhancer interactions (GeneHancer), and chromatin state tracks from craniofacial data [14]. (C) results from single-cell RNA sequencing of craniofacial tissue [15] including cell information (left) and gene expression levels for *CALD1* (right).

**Fig 4:**
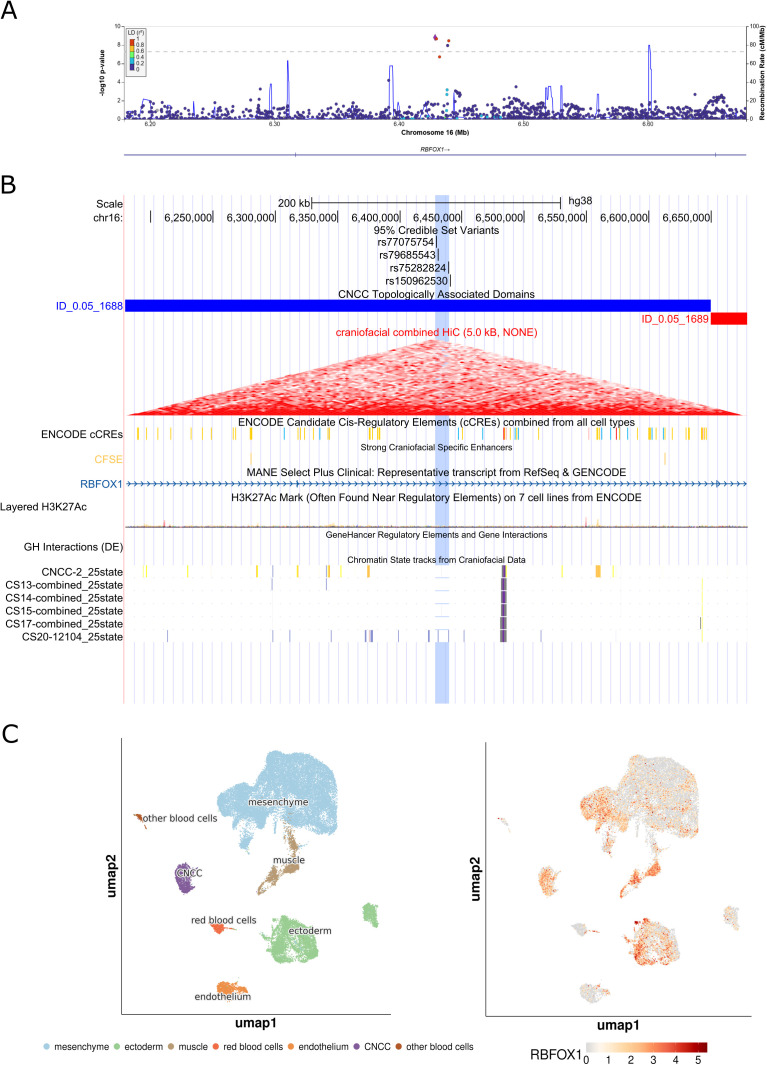
Integrated genomic analysis of the association at 16p13.3 with cleft lip with or without cleft palate (CL/P). (A) Regional association plots of meta-analyses for 16p13.3 with cleft lip and palate (CL/P) generated using LocusZoom [11]. Association results were obtained via fixed effects p-value based meta-analysis using METAL [12]. Variants are colored based on LD with the top variant in each region (shown in purple) based on the 1000 Genomes Phase 3 all populations references. (B) UCSC Genome Browser view, annotated with 95% credible set variants, cranial neural crest cell (CNCC) specific topologically associated domains (TADs) derived from craniofacial Hi-C data [13], candidate cis-regulatory elements from non-craniofacial cell types (ENCODE) as well as strong craniofacial specific enhancers [13], gene-enhancer interactions (GeneHancer), and chromatin state tracks from craniofacial data [14]. (C) results from single-cell RNA sequencing of craniofacial tissue [15] including cell information (left) and gene expression levels for *RBFOX1* (right).

We observed four novel associations at biologically plausible loci at 8q22.1, 1p36.32, 7q33, and 16p13.3. At the 8q22.1 locus, we observed an association between rs4735314 (chr8:94656529 T > C) with CLP and CL/P (p_CLP_ = 1.07e-9, p_CL/P_ = 3.88e-8). While the 8q22.1 locus has been implicated in prior studies, the signal we observed at this region was independent from what has been previously reported [10] and contained seven intronic variants in *ESRP1* (**Fig 1**). The previously identified variants at this locus (rs12681366 and rs957448) were 267 and 127 kb away, respectively, from the signal observed here and were not in linkage disequilibrium (r^2^ < 0.2) with associated variants in this study. Indeed, the 95% credible set for the association at 8q22.1 (rs67113852, chr8:94655603 G > T, p_CLP_ = 3.60e-9; rs11390715, chr8:94652479 G > GT, p_CLP_ = 5.94e-9; rs10089502, chr8:94651594 T > C, p_CLP_ = 2.20e-8; rs7826047, chr8:94699613 C > T, p_CLP_ = 1.29e-8; rs7845544, chr8:94704481 G > T, p_CLP_ = 2.73e-8; and rs10109430, chr8:94720538 A > G, p_CLP_ = 2.84e-9) did not include these previously reported variants. In addition, we also observed association signals at three novel loci, 1p36.32, 7q33, and 16p13.3. The 1p36.32 locus was associated with CLO (rs584402, chr1:5238225 C > T, p_CLO_ = 3.17e-8) and the 95% credible set at this locus included the lead variant (rs584402) and six additional variants (rs586165, chr1:5237827 T > C, p_CLO_ = 6.76e-8; rs679712, chr1:5238707 T > A, p_CLO_ = 4.77e-8; rs551536, chr1:5238999 T > C, p_CLO_ = 5.84e-8; rs551376, chr1:5239052 G > A, p_CLO_ = 4.77e-8; rs549773, chr1:5239163 C > A, p_CLO_ = 3.45e-8; rs5772197, chr1:5239601 ACT > A, p_CLO_ = 3.14e-8) which are all intergenic variants ~ 446 kb downstream of *AJAP1* (Fig 2). The 7q33 locus was associated with CLP (lead variant rs17168118, chr7:134892089 A > G, p_CLP_ = 9.17e-9) and the 95% credible set at this locus included the lead variant (rs17168118) and three additional variants (rs2075463, chr7:134867451 G > A, p_CLP_ = 7.48e-7; rs58715694, chr7:134874323 G > C, p_CLP_ = 6.17e-7; rs10488465, chr7:134877068 T > C, p_CLP_ = 2.29e-7), all intronic variants in *CALD1* (Fig 3). The third novel locus, at 16p13.3, was associated with CL/P and any cleft (lead variant rs77075754, chr16: 6429186 A > G, p_CL/P_ = 1.53e-9, p_ANY_ = 1.93e-9), which was driven by the GENEVA OFC cohort. The three additional SNPs that comprise the 95% credible set in this region (rs79685543, chr16:6430284 C > A, p_CL/P _= 2.11e-9; rs75282824, chr16:6439230 A>AT, p_CL/P _= 1.11e-8; rs150962530, chr16:6440126 G > A, p_CL/P _= 3.42e-9) are all contained within intron 2 of the MANE transcript of *RBFOX1* (Fig 4). The association at this locus was only observed in the GENEVA OFC cohort as the minor allele frequency (MAF) was below the 0.05 threshold used in analyses of the other two cohorts. The lead SNP at this locus is most common in populations with East Asian ancestry (gnomAD v4.1.0 East Asian MAF = 0.13, all other populations had MAF < 0.01). The direct examination of the lead SNP rs77075754 did not show evidence of association with CL/P in POFC1 (p_CL/P_ = 0.71, MAF = 0.013) or POFC2 (p_CL/P_ = 0.83, MAF = 0.033).

**Fig 5:**
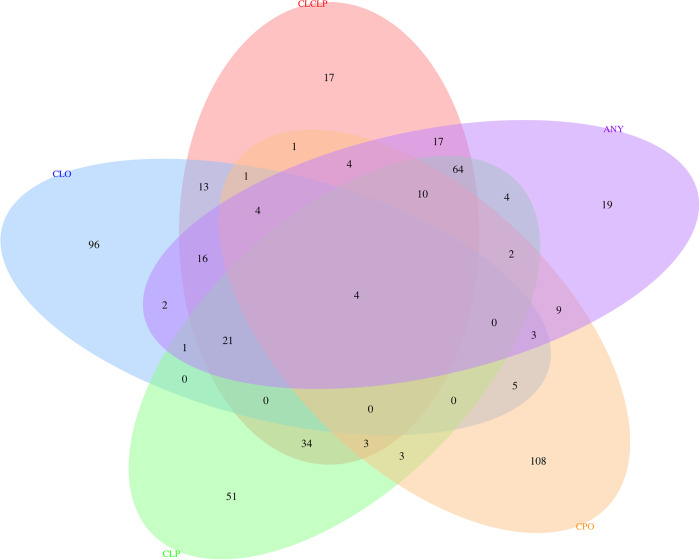
Venn Diagram showing the number of genes significant in TWAS results for the five cleft types. Colors indicate cleft type definition: ANY in purple, CL/P in orange, CLP in green, CLO in blue, and CPO in yellow. There were four genes associated with each cleft type definition (*PTPRS*, *C9orf170*, *RP1-40E16.9*, *RP11-87G24.6*).

**Fig 6:**
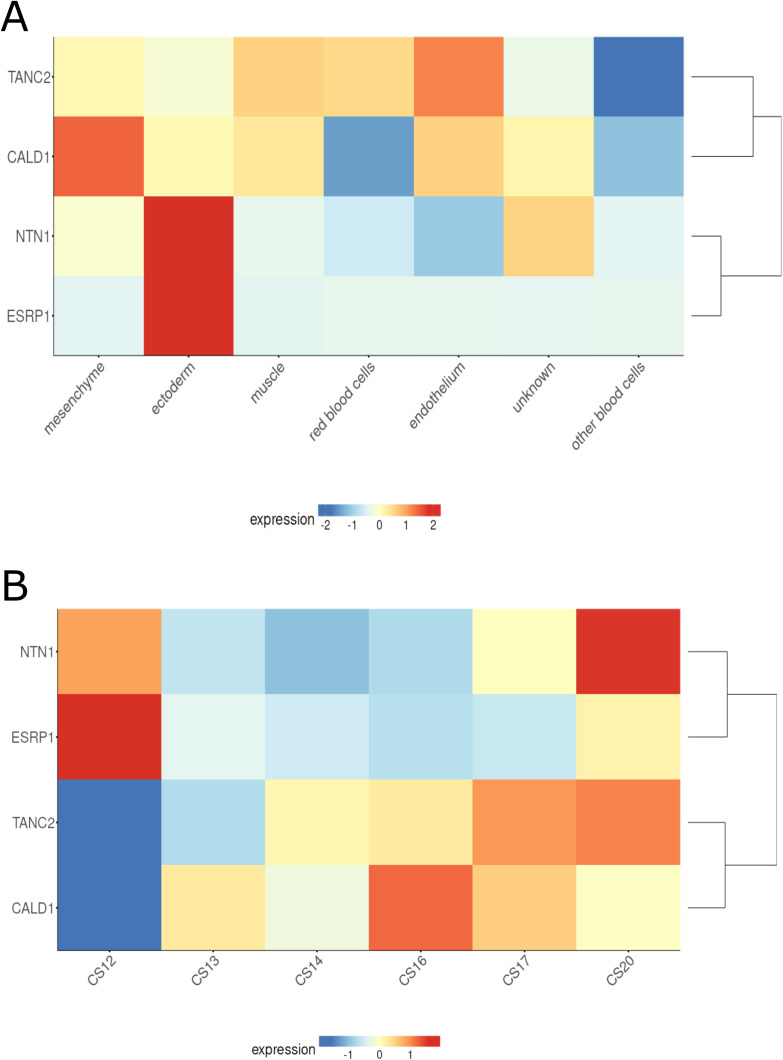
Gene expression patterns from single-cell RNA sequencing of human craniofacial tissue [15] of four genes intersecting all ancestry GWAS and TWAS (*CALD1*, *ESRP1*, *NTN1*, and *TANC2*) (A) grouped by cell type and (B) grouped by Carnegie Stage (CS). Normalized expression levels are averaged across group, log-transformed, and then scaled for comparability. Hierarchical clustering of genes based on expression patterns is shown.

We also examined predicted gene expression to identify genetic regulatory mechanisms associated with orofacial clefting. For each phenotype, we performed multi-tissue transcriptome-wide association studies (TWAS, Fig F - J in S1 Text) and examined the overlap with loci discovered in the genome-wide meta-analyses. We observed significant associations between genetically predicted expression and OFC risk for at least one OFC phenotype, implicating a total of 512 genes (S2 Table; Fig 5). Of these, four genes overlapped with a genome-wide significant signal from the meta-analysis and demonstrated significant association with genetically predicted expression and more than one OFC trait: *NTN1* (p_ANY_ = 1.66e-14, p_CL/P_ = 9.36e-20, p_CLP_ = 8.08e-25), *CALD1* (p_ANY_ = 2.925e-12, p_CL/P_ = 1.26e-15, p_CLP_ = 3.61e-16), *ESRP1* (p_ANY_ = 4.95e-8, p_CL/P_ = 7.92e-12, p_CLP_ = 6.89e-11)*, and TANC2* (p_CL/P_ = 1.27e-10, p_CLP_ = 2.24e-8). These genes exhibit temporal and cell-type specific expression patterns in human craniofacial tissue (Fig 6). We also performed gene set enrichment analysis of the 512 significant genes to identify significantly enriched biological processes, cellular components, and molecular function. We identified 10 significantly enriched biological processes including those relating to growth and development (GO:0040008, GO:0040007, GO:0048731) and response to stimuli (GO:0070887, GO:0050896) and one cellular component (cell periphery, GO:0071944) (Table 2).

**Table 2:** Significantly enriched Gene Ontology (GO) terms among the 512 genes showing significant results in the all ancestry TWAS. Enrichment analyses were conducted using the multi-query in g:Profiler [16]. Columns include source, term name, GO id, and adjusted p-values across each phenotype definition. P-values were adjusted to maintain an experiment-wide type 1 error rate of 0.05 using the g:SCS algorithm which accounts for the dependent structure of functionally annotated gene sets.

Source	Term name	Term id	adj p ANY	adj p CL/P	adj p CLP	adj p CLO	adj p CPO
GO:BP	multicellular organismal process	GO:0032501	0.00008	0.00001	0.00001	1	1
GO:BP	cellular response to chemical stimulus	GO:0070887	1	0.09717	0.00006	1	1
GO:BP	regulation of growth	GO:0040008	0.00341	0.00138	0.06788	1	1
GO:BP	growth	GO:0040007	0.03217	0.00180	0.01245	1	1
GO:BP	positive regulation of sarcomere organization	GO:0060298	1	1	1	1	0.00242
GO:BP	response to stimulus	GO:0050896	0.00624	0.00960	0.25129	1	1
GO:BP	system development	GO:0048731	0.00914	0.00788	0.00937	1	1
GO:BP	regulation of biological quality	GO:0065008	1	0.14047	0.01793	1	1
GO:BP	thyroid hormone generation	GO:0006590	1	1	0.02126	1	1
GO:BP	formation of primary germ layer	GO:0001704	1	0.04015	1	1	1
GO:CC	cell periphery	GO:0001704	0.02245	0.00003	0.06350	0.04255	1

Abbreviations GO: gene ontology, BP: biological process, CC: cellular component, ANY: any cleft, CL/P: cleft lip with/without cleft palate, CLP: cleft lip and palate, CLO: cleft lip only, CPO: cleft palate only.

### Ancestry-specific meta-analyses and TWAS

In the majority European ancestry meta-analyses, we observed four genome-wide significant loci (1p36.13 [PAX7], 8q21.3 [*DCAF4L2/MMP16*], 8q24.21 [*CCDC26*], and 17q23.2 [*TANC2]*) that have all been previously identified in association with OFC (S3-S4 Tables; Figs U–Y in S1 Text). In the majority Central/South American ancestry meta-analyses, we also observed two genome-wide significant loci (1q32.2 [*IRF6*] and 8q24.21 [*CCDC26*]) that have been previously identified (S6-S7 Tables; Figs AE–AI in S1 Text). No genome-wide significant signals were observed in the majority Asian ancestry nor majority African ancestry meta-analyses, likely reflecting low statistical power to due limited sample sizes.

The ancestry-specific TWAS results identified significant associations between genetically predicted gene expression and OFC risk in at least one OFC phenotype for 311, 20, 35, and 181 genes in the majority Asian (S9 Table), European (S5 Table), Central/South American (S8 Table), and African (S10 Table) ancestry groupings, respectively. None of these overlapped with genes observed at genome-wide significance in the meta-analyses. In ancestry-specific gene set enrichment analyses we identified significant enrichment of three molecular function terms (cytidine kinase activity, GO:0043771; uridine kinase activity, GO:0004849, and gamma-tubulin binding, GO:0043015) and one cellular component (side of membrane, GO:0098552) (S11 Table).

## Discussion

Through meta-analysis of >14,000 individuals and subsequent TWAS, we identified four novel loci associated with risk of nonsyndromic orofacial clefting on 1p36.32, 7q33, and 16p13.3, each pointing to biologically relevant candidate genes that play a role in embryonic craniofacial development (discussed below). We also identified a new independent signal and candidate gene, *ESRP1*, near a previously associated locus at 8q22.1. We also observed evidence for 12 known risk loci (1p36.13, 1p22.1, 1q32.2, 2p21, 5p12, 8q21.3, 8q24.21, 10q25.3, 13q31.1, 17p13.1, 17q23.2, and 20q12), many of which harbored multiple independent signals. Ancestry-specific meta-analyses did not yield any novel genome-wide significant loci, but recapitulated evidence for known cleft loci on 1q36.13, 1q32.2, 8q21.3, 8q24.21, and 17q23.2,

The association signal on 1p36.32 was uniquely seen in the GWAS for CLO and falls in a large intergenic region ~446 Kb downstream of *AJAP1* and ~753 Kb upstream of *KCNAB2* and is near a craniofacial specific enhancer element (Fig 2). *AJAP1* is a gene involved in the negative regulation of cell-matrix adhesion and wound healing [17] and is downregulated in human craniofacial tissues during CS14–22 compared to CS13 and control cells [13], and shows little expression in craniofacial tissue at CS22 (Fig 2). *KCNAB2* is a known gene involved in 1p36 deletion syndrome, which is characterized by distinct craniofacial features including microbrachycephaly and can include orofacial clefts [18,19]. This novel association may highlight the regulatory role of this region (either through *AJAP1*, *KCNAB2*, or both) in the development of cleft lip.

The 7q33 locus is spanned by *CALD1* (Caldesmon 1), a gene encoding a calmodulin- and actin-binding protein that plays an essential role in the regulation of smooth muscle and nonmuscle contraction [17]. Preclinical models suggest Cald1 has a role in building morphology through blood vessel endothelial cell migration and tube morphogenesis [20–23]. Caldesmon is highly expressed in pre-migratory and migrating cranial neural crest cells in xenopus embryos, and its depletion causes these cells to migrate a significantly shorter distance [24]. CALD1 is also highly expressed in human and murine craniofacial tissue across CS13–20/E8.5-10.5, during which the formation of the lip and palate occurs [13,25]. The association signal in this region is led by a cross-tissue eQTL for *CALD1*, with the G allele being simultaneously associated with greater OFC risk (this study) and decreased *CALD1* expression (GTEx Portal accessed July 3, 2025).This association signal also directly overlaps a known craniofacial super-enhancer as well as 6 other super-enhancers (aorta, duodenum smooth muscle, osteoblast, myotube, stomach smooth muscle and u87 cells) affecting *CALD1* expression [14]. *CALD1* is also highly expressed in many craniofacial tissue cell types (Fig 3). Together, this suggests that CALD1 plays an important role in embryonic craniofacial development, likely through regulating neural crest cell adhesion, spread, and motility by modulating actin dynamics.

The signal on 16p13.3 is contained between two recombination hotspots located within a much larger topologically associated domain spanning the first two introns of *RBFOX1*. *RBFOX1* encodes an RNA binding protein and regulates alternative splicing of transcripts [17]. In humans, *RBFOX1* is expressed in neurons, heart, and muscle [26] as well as within muscle precursor cells from human craniofacial tissue [13]. *RBFOX1* is highly conserved and predicted intolerant to loss of function (loeuf 0.31) [27]. In mice, *Rbfox1* was differentially expressed during neural tube development (downregulated at E9.5, during the fusion of the neural tube) [28]. However, some caution should be taken in this interpretation, as the association at this locus was only observed in the GENEVA OFC cohort, likely due to the larger number of participants of East Asian ancestry, and not observed in the majority Asian ancestry subpopulation analysis, so replication is still needed for this locus.

Association with orofacial clefting at the 8q22.1 locus has been previously reported for two SNPs: rs957448 (chr8:94529074 A > G) an intronic SNP in *VIRMA*, and rs12681366 (chr8: 94389037 T > C) an intronic SNP in *RAD54B* [10]. In our analysis, we identified a separate signal led by rs4735314 not in LD with these two previously reported variants (LD r^2^ < 0.2). This new signal entirely comprises intronic SNPs in *ESRP1*, a gene that was also nominated through the multi-tissue TWAS. Interestingly, each of these three variants are significant eQTLs for both *ESRP1* and a divergent transcript of *VIRMA* (*VIRMA-DT*, ENSG00000253704.3) in some tissues [26]. *ESRP1* and its paralog *ESRP2* are epithelial cell-type-specific splicing regulators that regulate craniofacial development and the splicing of craniofacial and cleft associated genes including *CTNND1* and the FGFR2 signaling pathway [17,29,30]. Mutations in *ESRP2* and *ESRP1/2* splicing target *CTNND1* are associated with orofacial clefts [30,31]. In addition, mutations in *FGFR2* and other FGF genes lead to craniosynostosis, syndactyly, and cleft palate [32–36], demonstrating the potential importance of *ESRP1* to cleft risk. *ESRP1* and *VIRMA* are targets for known super enhancers (from colon crypt tissue, gastric tissue, NHEK cells, HCC1954 cells, ACO 9m cells) as well as craniofacial specific enhancers [14,37]. In mice, *Esrp1* has been established as an epithelial-specific splicing factor, the ablation of which leads to fully penetrant bilateral CL/P [38], regulating *Fgfr2* splicing and expression of canonical Wnt signaling genes, including *Wnt9b* and *Shh* [39]. Within human craniofacial tissue, *ESRP1* is solely expressed in ectodermal cells (**Fig 1**), and in bulk RNA-seq shows differential expression at critical stages for facial formation (CS13, CS14, CS15, CS17, and CS20) [13]. *RAD54B*, the gene suggested by a previous study [40,41], is also differentially expressed in human craniofacial tissue across the period critical to facial development like *ESRP1*, but is not cell-sub-type-specific in its expression [13]. *VIRMA*, another candidate gene in the region, is expressed in human craniofacial tissue but is not differentially expressed during the critical period of lip and palate formation [13]. Together, this evidence supports *ESRP1* as a strong orofacial cleft risk locus warranting further functional investigation.

Through TWAS, we identified numerous genes with potential regulatory roles in OFC risk, many overlapping with GWAS signals. However, a limitation of this TWAS is the lack of relevant tissue with which to evaluate expression patterns; these results may exclude tissue-specific expression that is exclusive to embryonic tissue and/or craniofacial tissue. These TWAS results were enriched for many biological processes including those involved in growth and response to stimuli. These findings reflect that OFC risk is genetically regulated through several mechanisms—both those that directly regulate embryonic growth and those that respond to certain in-utero environmental exposures. The TWAS results additionally supported the potential regulatory role of *CALD1* and *ESRP1* in association with clefting as well as two known genes associated with OFC-risk, *NTN1* and *TANC2* [42,43]. As described above, *CALD1* expression likely regulates neural crest cell migration critical for embryonic craniofacial development, while *ESRP1* likely affects craniofacial development through regulation of splicing of critical pathways for craniofacial development. Prior studies have nominated a potential role in orofacial cleft risk for *NTN1* (Netrin 1) in palatal fusion through regulation of cell migration in embryogenesis and other developmental processes [42,44–52]. The role of *TANC2* in OFC is still unknown. TANC2 is a scaffolding protein that is predicted to act upstream of or within embryonic development [17]; it is highly expressed in the brain in adults (GTEx portal accessed July 3, 2025) but also in human craniofacial tissue [15]. Mutations in *TANC2* cause neurodevelopmental and psychiatric disorders [53–57], including a case presenting with craniofacial dysmorphic features [54].

As expected, nearly every locus we identified demonstrated genome-wide significance with CL/P, recapitulating the evidence for shared genetic architecture between CLP and CLO for many loci. However, this was not the case for the novel associations with the *AJAP1/KCNAB2* (1p36.32) locus, which was only seen in CLO alone, and with *CALD1*, which only surpassed genome-wide significance when examining CLP alone, and not combining it with CLO as a composite phenotype as is typically done to maximize statistical power. While the *CALD1* locus still demonstrated association with the composite phenotype CL/P, this example highlights the importance of examining subtype-specific associations to maximize power to detect loci enriched for association within one phenotype definition. None of the loci observed in this study were associated with CPO, despite prior evidence of association with *GRHL3* in a subset of the individuals used in this meta-analysis, all of European genetic ancestry [58].

In summary, this study provides strong evidence for two novel genes (*CALD1* at 7q33 and *ESRP1* at 8q22.1), in association with orofacial cleft risk through transcriptomic and genomic analyses. These loci are each associated with many orofacial cleft phenotypes (CLO, CLP, CL/P, and/or any cleft) and are regulated by both craniofacial-specific and cross-tissue super enhancers, suggesting that they may represent more global regulators of embryonic growth and development beyond their role in facial formation. This study also demonstrates the utility of multi-tissue TWAS and colocalization for identifying these putative regulators, despite the absence of a relevant tissue type in existing eQTL databases.

## Methods

### Ethics statement

Written informed consent was obtained for all participants and ethical approval was obtained for all sites: The University of Pittsburgh of the Commonwealth System Higher Education, Institutional Review Board (IRB) (FWA00006790), [Coordinating Center and Pennsylvania sites approval]; The University of Iowa IRB (FWA00003007), [Iowa sites approval]; Johns Hopkins University IRB (FWA00005834); University of Texas Houston Health Sciences IRB (FWA00000667), [Texas sites approval]; University of Puerto Rico, Medical Sciences Ethical Committee (FWA00005561), [Puerto Rico site approval]; Ethics Committee of Fundacion Clinica Noel de Medellin, Colombia; Ethics Committees of the Hospital Nacional de Huehuetenango and Hospital Nacional Tiquisate, Guatemala; GAT Foundation, Budapest, Hungary; Lagos University Teaching Hospital, Health Research Ethics Committee, Lagos, Nigeria; ECLAMC/CEMIC Ethics Committees (FWA00001566), Buenos Aires, Argentina; and University of the Philippines, Manila, Ethics Committee (FWA00003505).

### Samples and genotyping

Samples and data for these analyses were derived from three studies of orofacial clefts and are summarized in Table 3. The first, referred to here as GENEVA OFC, comprised 5,856 individuals from 1,953 case-parent trios of isolated, nonsyndromic orofacial clefts that were recruited from several sites across the United States, Western Europe (Norway, Denmark), and East Asia (Korea, Singapore, Taiwan, Philippines, and China) as part of the International Consortium to Identify Genes and Interactions Controlling Oral Clefts, part of the Gene Environment Association Studies (GENEVA) initiative (dbGaP accession number phs000094.v1.p1). For the purposes of running ancestry-specific meta-analyses, ancestry groupings in GENEVA OFC were determined by recruitment site and was limited to European- and Asian-majority ancestry groups for this cohort (Table 3). More details about recruiting these participants and genotyping QC have been described elsewhere [59]. Briefly, samples were genotyped for 589,945 SNPs on the Illumina Human610-Quadv.1_B BeadChip. A total of 412 overlapping individuals who were re-genotyped in subsequent studies were excluded from the analysis of GENEVA OFC herein.

**Table 3:** Sample size by phenotype and ancestry group.

Ancestry Group	Cohort	Controls	CLO	CPO	CLP	CL/P	ANY ^a^
All	GENEVA OFC ^b^	---	431	455	1064	1495	1953
	POFC1	2620	624	299	1946	2570	2881
	POFC2	1503	469	172	980	1449	1621
Asian	GENEVA OFC ^b^	---	215	237	680	895	1135
	POFC1	158	204	43	327	531	574
	POFC2	558	294	66	699	993	1059
European	GENEVA OFC ^b^	---	210	204	369	579	783
	POFC1	1300	173	181	501	674	856
	POFC2	154	35	20	95	130	150
Central/South American	POFC1	1072	189	52	1026	1215	1273
	POFC2	168	17	0	52	69	69
African	POFC1	79	57	23	90	147	175
	POFC2	623	123	86	134	257	343

^a^includes some cases with unknown cleft sub-type.

^b^number of trios.

Abbreviations ANY: any cleft, CL/P: cleft lip with/without cleft palate, CLP: cleft lip and palate, CLO: cleft lip only, CPO: cleft palate only.

The second study, referred to here as POFC1, included 2,881 cases and 2,620 controls (unaffected individuals from families with no reported history of OFCs) from the Genetics of Orofacial Clefts and Related Phenotypes study (dbGaP accession number phs000774.v2.p1). Participants were recruited from 13 countries in North America (United States), Central or South America (Guatemala, Argentina, Colombia, Puerto Rico), Asia (China, Philippines), Europe (Denmark, Turkey, Spain), and Africa (Ethiopia, Nigeria). Samples were genotyped for 539,473 SNPs on the Illumina HumanCore+Exome array. Ancestry grouping in POFC1 was determined through examining Principal Components of Ancestry (PCA) as described previously (Table 3) [43]. Additional details on recruitment, genotyping, and quality controls have been previously described [43,58].

The third study, referred to here as POFC2, included 1,621 cases and 1,503 controls from the Genetics of Orofacial Clefts, Sub-types, and Subclinical Phenotypes study (dbGaP accession number phs002815.v2.p1). K-means clustering of the first 3 PCAs was used to define ancestry groupings in POFC2 (Table 3; Fig AY in S1 Text). Genotyping was performed at CIDR using the Infinium Global Diversity Array-8 v1.0 Array along with extensive quality control following the methods of Laurie et al.[60] yielding 1,434,549 SNPs.

For each study, we imputed genotypes with the TOPMed reference panel via the TOPMed Imputation Server [61]. We removed variants with poor imputation quality (r^2^ < 0.80) and extracted the most likely genotype, keeping only those with a minimum genotype probability of 0.9. Prior to association testing we also removed variants with Mendelian error rates >0.5% [in GENEVA OFC only], missing call rate >5%, and minor allele frequency (MAF) <5%.

We then performed analyses for five phenotype definitions: CLO, CPO, CLP, CL/P, and ANY. These analyses were repeated across ancestry groups: All, majority Asian ancestry, majority European ancestry, majority Central/South American ancestry, and majority African ancestry.

### Genome-wide association analysis

For each phenotype, we analyzed the case-parent trios in GENEVA OFC with the transmission/disequilibrium test (TDT) as implemented in PLINK [62]. For the POFC1 and POFC2 studies, we performed separate mixed logistic regression models using an additive genotype model as implemented in GENESIS, with a saddle point p-value approximation to account for case/control sample size imbalance and using a genetic relatedness matrix derived from PC-Relate and adjusting for fixed effects of age, sex, and principal components of ancestry (PCAs), 10 and 8 for POFC1 and POFC2 respectively, derived with PC-AiR [63,64]. Results were combined across the three studies using a p-value-based meta-analysis with METAL [12]. Regional association plots and credible sets for novel loci were generated using LocusZoom [11]. We annotated variants and identified independent lead SNPs (those with meta-analysis p < 5e-8 and LD r^2^ < 0.1 with another lead SNP) among associated loci from the meta-analysis summary statistics with FUMA [65].

### Multi-tissue transcriptome-wide association analysis

Using S-PrediXcan [66], we combined cis-eQTL data from all tissues in the GTEx Project (version 8) [26] with our meta-analysis summary statistics using pre-specified weights based on covariance of genetic variants from PredictDB [67]. We then combined S-PrediXcan data from all tissues and jointly analyzed them with S-MulTiXcan [68]. We used a Bonferroni-adjusted significance threshold of 2.29e-6 (i.e., 0.05/21838) to account for the number of genes tested. Gene set enrichment analyses of the significant genes identified in TWAS was performed using g:Profiler using a multiple query (separate gene lists for each phenotype definition) and using an adjusted p-value to maintain an experiment-wide type 1 error rate of 0.05 with the g:SCS algorithm that accounts for dependencies among gene annotations [16].

## Supporting information

S1 Table Association results for all genome-wide significant variants (p < 5e-8) from all ancestry meta-analysis. Columns include chromosome (Chr), position in hg38 (Pos), reference and alternate (effect) alleles (Ref/Alt), rsID, nearest gene, alternate allele frequency (AAF) for POFC1, POFC2, and GENEVA OFC, p-values and direction of effects for POFC1, POFC2, and GENEVA OFC, respectively (? indicates variant was not present in that study) for the five cleft phenotypes. Abbreviations Any: any cleft, CL/P: cleft lip with/without cleft palate, CLP: cleft lip and palate, CLO: cleft lip only, CPO: cleft palate only.
(XLSX)

S2 Table Results from all ancestry multi-tissue TWAS for each gene surpassing the significance threshold (p < 2.29e-6) in at least one phenotype. Columns include Ensemble gene id, gene name, and p-values from the multi-tissue TWAS by each phenotype. Abbreviations: ANY = any cleft, CL/P = cleft lip with/without cleft palate, CLP = cleft lip and palate, CLO = isolated cleft lip, CPO = isolated cleft palate.
(XLSX)

S3 Table Results from genome-wide European ancestry meta-analysis for each independent lead variant demonstrating genome-wide significance (p < 5e-8) in at least one phenotype. Columns include chromosome (Chr), position in hg38 (Pos), reference and alternate (effect) alleles (Ref/Alt), nearest gene, alternate allele frequency (AAF) average, standard error, and min/max across POFC1, POFC2, and GENEVA OFC, p-values and direction of effects for POFC1, POFC2, and GENEVA OFC, respectively (? indicates variant was not present in that study) for the five cleft phenotypes. Abbreviations Any: any cleft, CL/P: cleft lip with/without cleft palate, CLP: cleft lip and palate, CLO: cleft lip only, CPO: cleft palate only.
(XLSX)

S4 Table Association results for all genome-wide significant variants (p < 5e-8) from European ancestry meta-analysis. Columns include chromosome (Chr), position in hg38 (Pos), reference and alternate (effect) alleles (Ref/Alt), nearest gene, alternate allele frequency (AAF) average, standard error, and min/max across POFC1, POFC2, and GENEVA OFC, p-values and direction of effects for POFC1, POFC2, and GENEVA OFC, respectively (? indicates variant was not present in that study) for the five cleft phenotypes. Abbreviations Any: any cleft, CL/P: cleft lip with/without cleft palate, CLP: cleft lip and palate, CLO: cleft lip only, CPO: cleft palate only.
(XLSX)

S5 Table Results from European ancestry multi-tissue TWAS for each gene surpassing the significance threshold (p < 2.29e-6) in at least one phenotype. Columns include Ensemble gene id, gene name, and p-values from the multi-tissue TWAS by each phenotype. Abbreviations: ANY = any cleft, CL/P = cleft lip with/without cleft palate, CLP = cleft lip and palate, CLO = isolated cleft lip, CPO = isolated cleft palate.
(XLSX)

S6 Table Results from genome-wide Central/South American ancestry meta-analysis for each independent lead variant demonstrating genome-wide significance (p < 5e-8) in at least one phenotype. Columns include chromosome (Chr), position in hg38 (Pos), reference and alternate (effect) alleles (Ref/Alt), nearest gene, alternate allele frequency (AAF) average, standard error, and min/max across POFC1 and POFC2, p-values and direction of effects for POFC1 and POFC2, respectively (? indicates variant was not present in that study) for the five cleft phenotypes. Abbreviations Any: any cleft, CL/P: cleft lip with/without cleft palate, CLP: cleft lip and palate, CLO: cleft lip only, CPO: cleft palate only.
(XLSX)

S7 Table Association results for all genome-wide significant variants (p < 5e-8) from Central/South American ancestry meta-analysis. Columns include chromosome (Chr), position in hg38 (Pos), reference and alternate (effect) alleles (Ref/Alt), nearest gene, alternate allele frequency (AAF) average, standard error, and min/max across POFC1 and POFC2, p-values and direction of effects for POFC1 and POFC2, respectively (? indicates variant was not present in that study) for the five cleft phenotypes. Abbreviations Any: any cleft, CL/P: cleft lip with/without cleft palate, CLP: cleft lip and palate, CLO: cleft lip only, CPO: cleft palate only.
(XLSX)

S8 Table Results from Central/South American ancestry multi-tissue TWAS for each gene surpassing the significance threshold (p < 2.29e-6) in at least one phenotype. Columns include Ensemble gene id, gene name, and p-values from the multi-tissue TWAS by each phenotype. Abbreviations: ANY = any cleft, CL/P = cleft lip with/without cleft palate, CLP = cleft lip and palate, CLO = isolated cleft lip, CPO = isolated cleft palate.
(XLSX)

S9 Table Results from Asian ancestry multi-tissue TWAS for each gene surpassing the significance threshold (p < 2.29e-6) in at least one phenotype. Columns include Ensemble gene id, gene name, and p-values from the multi-tissue TWAS by each phenotype. Abbreviations: ANY = any cleft, CL/P = cleft lip with/without cleft palate, CLP = cleft lip and palate, CLO = isolated cleft lip, CPO = isolated cleft palate.
(XLSX)

S10 Table Results from African ancestry multi-tissue TWAS for each gene surpassing the significance threshold (p < 2.29e-6) in at least one phenotype. Columns include Ensemble gene id, gene name, and p-values from the multi-tissue TWAS by each phenotype. Abbreviations: ANY = any cleft, CL/P = cleft lip with/without cleft palate, CLP = cleft lip and palate, CLO = isolated cleft lip, CPO = isolated cleft palate.
(XLSX)

S11 Table Significantly enriched Gene Ontology (GO) terms among the genes showing significant results in the ancestry-specific TWAS. Enrichment analyses were conducted separately for each ancestry grouping using the multi-query in g:Profiler. Columns include ancestry group, source, term name, GO id, and adjusted p-values across each phenotype definition. P-values were adjusted to maintain an experiment-wide type 1 error rate of 0.05 within ancestry group using the g:SCS algorithm which accounts for the dependent structure of functionally annotated gene sets.
(XLSX)

S1 Text Results from meta-analysis and TWAS. (DOCX)

## References

[1] Dixon MJ, Marazita ML, Beaty TH, Murray JC. Cleft lip and palate: understanding genetic and environmental influences. Nat Rev Genet. 2011;12(3):167–78. doi: 10.1038/nrg2933 21331089 PMC3086810

[2] Hammond NL, Dixon MJ. Revisiting the embryogenesis of lip and palate development. Oral Dis. 2022;28(5):1306–26. doi: 10.1111/odi.14174 35226783 PMC10234451

[3] Leslie EJ, Marazita ML. Genetics of cleft lip and cleft palate. Am J Med Genet C Semin Med Genet. 2013;163C(4):246–58. doi: 10.1002/ajmg.c.31381 24124047 PMC3925974

[4] Rahimov F, Jugessur A, Murray JC. Genetics of nonsyndromic orofacial clefts. Cleft Palate Craniofac J. 2012;49(1):73–91. doi: 10.1597/10-178 21545302 PMC3437188

[5] Uribe LMM, Marazita ML. Epidemiology, etiology, and genetics of orofacial clefting. Cleft and Craniofacial Orthodontics. Wiley. 2022. 39–60. doi: 10.1002/9781119778387.ch4

[6] Alade A, Awotoye W, Butali A. Genetic and epigenetic studies in non-syndromic oral clefts. Oral Dis. 2022;28(5):1339–50. doi: 10.1111/odi.14146 35122708

[7] Leslie EJ, Carlson JC, Shaffer JR, Buxó CJ, Castilla EE, Christensen K, et al. Association studies of low-frequency coding variants in nonsyndromic cleft lip with or without cleft palate. Am J Med Genet A. 2017;173(6):1531–8. doi: 10.1002/ajmg.a.38210 28425186 PMC5444956

[8] Butali A, Adeyemo WL, Mossey PA, Olasoji HO, Onah II, Adebola A, et al. Prevalence of orofacial clefts in Nigeria. Cleft Palate Craniofac J. 2014;51(3):320–5. doi: 10.1597/12-135 23557093 PMC3706513

[9] IPDTOC Working Group. Prevalence at birth of cleft lip with or without cleft palate: data from the International Perinatal Database of Typical Oral Clefts (IPDTOC). Cleft Palate Craniofac J. 2011;48(1):66–81. doi: 10.1597/09-217 20507242

[10] Yu Y, Zuo X, He M, Gao J, Fu Y, Qin C, et al. Genome-wide analyses of non-syndromic cleft lip with palate identify 14 novel loci and genetic heterogeneity. Nat Commun. 2017;8:14364. doi: 10.1038/ncomms14364 28232668 PMC5333091

[11] Boughton AP, Welch RP, Flickinger M, VandeHaar P, Taliun D, Abecasis GR. LocusZoom.js: Interactive and embeddable visualization of genetic association study results. Bioinformatics. 2021. doi: 10.1093/bioinformatics/btab186 PMC8479674 33734315

[12] Willer CJ, Li Y, Abecasis GR. METAL: fast and efficient meta-analysis of genomewide association scans. Bioinformatics. 2010;26(17):2190–1. doi: 10.1093/bioinformatics/btq340 20616382 PMC2922887

[13] Yankee TN, Oh S, Winchester EW, Wilderman A, Robinson K, Gordon T, et al. Integrative analysis of transcriptome dynamics during human craniofacial development identifies candidate disease genes. Nat Commun. 2023;14(1):4623. doi: 10.1038/s41467-023-40363-1 37532691 PMC10397224

[14] Wilderman A, Van Oudenhove J, Kron J, Noonan JP, Cotney J. High-resolution epigenomic atlas of human embryonic craniofacial development. Cell Reports. 2018;23(5):1581–97. doi: 10.1016/j.celrep.2018.04.014 29719267 PMC5965702

[15] Khouri-Farah N, Winchester EW, Schilder BM, Robinson K, Curtis SW, Skene NG, et al. Gene expression patterns of the developing human face at single cell resolution reveal cell type contributions to normal facial variation and disease risk. bioRxiv. 2025. 2025.01.18.633396. https://www.biorxiv.org/content/10.1101/2025.01.18.633396v1

[16] Kolberg L, Raudvere U, Kuzmin I, Adler P, Vilo J, Peterson H. g:Profiler-interoperable web service for functional enrichment analysis and gene identifier mapping (2023 update). Nucleic Acids Res. 2023;51(W1):W207-12. 10.1093/nar/gkad347 PMC10320099 37144459

[17] Stelzer G, Rosen N, Plaschkes I, Zimmerman S, Twik M, Fishilevich S, et al. The GeneCards Suite: From Gene Data Mining to Disease Genome Sequence Analyses. Curr Protoc Bioinforma. 2016;54:1.30.1-1.30.33. 10.1002/cpbi.5 27322403

[18] Jordan VK, Zaveri HP, Scott DA. 1p36 deletion syndrome: an update. Appl Clin Genet. 2015;8:189–200. doi: 10.2147/TACG.S65698 26345236 PMC4555966

[19] Shimada S, Shimojima K, Okamoto N, Sangu N, Hirasawa K, Matsuo M, et al. Microarray analysis of 50 patients reveals the critical chromosomal regions responsible for 1p36 deletion syndrome-related complications. Brain Dev. 2015;37(5):515–26. doi: 10.1016/j.braindev.2014.08.002 25172301

[20] Abrams J, Davuluri G, Seiler C, Pack M. Smooth muscle caldesmon modulates peristalsis in the wild type and non-innervated zebrafish intestine. Neurogastroenterol Motil. 2012;24(3):288–99. doi: 10.1111/j.1365-2982.2011.01844.x 22316291 PMC3919438

[21] Zheng P-P, Severijnen L-A, van der Weiden M, Willemsen R, Kros JM. A crucial role of caldesmon in vascular development in vivo. Cardiovasc Res. 2009;81(2):362–9. doi: 10.1093/cvr/cvn294 18980955

[22] Zheng PP, Severijnen LA, Willemsen R, Kros JM. Caldesmon is essential for cardiac morphogenesis and function: in vivo study using a zebrafish model. Biochem Biophys Res Commun. 2009;378(1):37–40. 19000902 10.1016/j.bbrc.2008.10.165

[23] Zheng P-P, Severijnen L-A, Willemsen R, Kros JM. Images. Different patterns of circulatory shunting in zebrafish caldesmon morphants: a digital motion analysis. Heart Lung Circ. 2010;19(4):251–2. doi: 10.1016/j.hlc.2009.08.007 19914132

[24] Nie S, Kee Y, Bronner-Fraser M. Caldesmon regulates actin dynamics to influence cranial neural crest migration in Xenopus. Mol Biol Cell. 2011;22(18):3355–65. doi: 10.1091/mbc.E11-02-0165 21795398 PMC3172261

[25] Brunskill EW, Potter AS, Distasio A, Dexheimer P, Plassard A, Aronow BJ, et al. A gene expression atlas of early craniofacial development. Dev Biol. 2014;391(2):133–46. doi: 10.1016/j.ydbio.2014.04.016 24780627 PMC4095820

[26] The GTEx Consortium. The GTEx Consortium atlas of genetic regulatory effects across human tissues. Science. 2020;369(6509):1318–30. 32913098 10.1126/science.aaz1776 PMC7737656

[27] Karczewski KJ, Francioli LC, Tiao G, Cummings BB, Alföldi J, Wang Q, et al. The mutational constraint spectrum quantified from variation in 141,456 humans. Nature. 2020;581(7809):434–43. doi: 10.1038/s41586-020-2308-7 32461654 PMC7334197

[28] Yu J, Mu J, Guo Q, Yang L, Zhang J, Liu Z, et al. Transcriptomic profile analysis of mouse neural tube development by RNA-Seq. IUBMB Life. 2017;69(9):706–19. doi: 10.1002/iub.1653 28691208

[29] Carroll SH, Macias Trevino C, Li EB, Kawasaki K, Myers N, Hallett SA. An Irf6-Esrp1/2 regulatory axis controls midface morphogenesis in vertebrates. Dev Camb Engl. 2020;147(24):dev194498. doi: 10.1242/dev.194498 PMC7774891 33234718

[30] Caetano da Silva C, Macias Trevino C, Mitchell J, Murali H, Tsimbal C, Dalessandro E, et al. Functional analysis of ESRP1/2 gene variants and CTNND1 isoforms in orofacial cleft pathogenesis. Commun Biol. 2024;7(1):1040. doi: 10.1038/s42003-024-06715-3 39179789 PMC11344038

[31] Cox LL, Cox TC, Moreno Uribe LM, Zhu Y, Richter CT, Nidey N. Mutations in the Epithelial Cadherin-p120-Catenin Complex Cause Mendelian Non-Syndromic Cleft Lip with or without Cleft Palate. Am J Hum Genet. 2018;102(6):1143–57. 29805042 10.1016/j.ajhg.2018.04.009 PMC5992119

[32] Eswarakumar VP, Horowitz MC, Locklin R, Morriss-Kay GM, Lonai P. A gain-of-function mutation of Fgfr2c demonstrates the roles of this receptor variant in osteogenesis. Proc Natl Acad Sci U S A. 2004;101(34):12555–60. 15316116 10.1073/pnas.0405031101 PMC515096

[33] Passos-Bueno MR, Wilcox WR, Jabs EW, Sertié AL, Alonso LG, Kitoh H. Clinical spectrum of fibroblast growth factor receptor mutations. Hum Mutat. 1999;14(2):115–25. doi: 10.1002/(SICI)1098-1004(1999)14:2<115::AID-HUMU3>3.0.CO;2-2 10425034

[34] Rice R, Spencer-Dene B, Connor EC, Gritli-Linde A, McMahon AP, Dickson C, et al. Disruption of Fgf10/Fgfr2b-coordinated epithelial-mesenchymal interactions causes cleft palate. J Clin Invest. 2004;113(12):1692–700. doi: 10.1172/JCI20384 15199404 PMC420504

[35] Pauws E, Stanier P. FGF signalling and SUMO modification: new players in the aetiology of cleft lip and/or palate. Trends Genet. 2007;23(12):631–40. doi: 10.1016/j.tig.2007.09.002 17981355

[36] Riley BM, Mansilla MA, Ma J, Daack-Hirsch S, Maher BS, Raffensperger LM, et al. Impaired FGF signaling contributes to cleft lip and palate. Proc Natl Acad Sci U S A. 2007;104(11):4512–7. doi: 10.1073/pnas.0607956104 17360555 PMC1810508

[37] Fishilevich S, Nudel R, Rappaport N, Hadar R, Plaschkes I, Iny Stein T, et al. GeneHancer: genome-wide integration of enhancers and target genes in GeneCards. Database (Oxford). 2017;2017:bax028. doi: 10.1093/database/bax028 28605766 PMC5467550

[38] Bebee TW, Park JW, Sheridan KI, Warzecha CC, Cieply BW, Rohacek AM, et al. The splicing regulators Esrp1 and Esrp2 direct an epithelial splicing program essential for mammalian development. Elife. 2015;4:e08954. doi: 10.7554/eLife.08954 26371508 PMC4566030

[39] Lee S, Sears MJ, Zhang Z, Li H, Salhab I, Krebs P, et al. Cleft lip and cleft palate in Esrp1 knockout mice is associated with alterations in epithelial-mesenchymal crosstalk. Dev Camb Engl. 2020;147(21):dev187369. doi: 10.1242/dev.187369 PMC7225129 32253237

[40] Liu X, Yang S, Meng L, Chen C, Hui X, Jiang Y, et al. Association between PTCH1 and RAD54B single-nucleotide polymorphisms and non-syndromic orofacial clefts in a northern Chinese population. J Gene Med. 2018;20(12):e3055. doi: 10.1002/jgm.3055 30172247

[41] Morvaridi Farimani R, Azimi-Nezhad M, KhorramKhorshid HR, Ebadifar A, Tohidkhah S, Jafarian Z, et al. Association between PTCH1 and RAD54B Single-Nucleotide Polymorphisms and Non-syndromic Orofacial Clefts in the Northeast Population of Iran. Avicenna J Med Biotechnol. 2022;14(4):310–6. 36504563 PMC9706251

[42] Leslie EJ, Taub MA, Liu H, Steinberg KM, Koboldt DC, Zhang Q. Identification of functional variants for cleft lip with or without cleft palate in or near PAX7, FGFR2, and NOG by targeted sequencing of GWAS loci. Am J Hum Genet. 2015;96(3):397–411. 25704602 10.1016/j.ajhg.2015.01.004 PMC4375420

[43] Leslie EJ, Carlson JC, Shaffer JR, Feingold E, Wehby G, Laurie CA, et al. A multi-ethnic genome-wide association study identifies novel loci for non-syndromic cleft lip with or without cleft palate on 2p24.2, 17q23 and 19q13. Hum Mol Genet. 2016;25(13):2862–72. doi: 10.1093/hmg/ddw104 27033726 PMC5181632

[44] Serafini T, Colamarino SA, Leonardo ED, Wang H, Beddington R, Skarnes WC. Netrin-1 is required for commissural axon guidance in the developing vertebrate nervous system. Cell. 1996;87(6):1001–14. 8978605 10.1016/s0092-8674(00)81795-x

[45] Salminen M, Meyer BI, Bober E, Gruss P. Netrin 1 is required for semicircular canal formation in the mouse inner ear. Development. 2000;127(1):13–22. doi: 10.1242/dev.127.1.13 10654596

[46] Srinivasan K, Strickland P, Valdes A, Shin GC, Hinck L. Netrin-1/neogenin interaction stabilizes multipotent progenitor cap cells during mammary gland morphogenesis. Dev Cell. 2003;4(3):371–82. doi: 10.1016/s1534-5807(03)00054-6 12636918

[47] Park KW, Crouse D, Lee M, Karnik SK, Sorensen LK, Murphy KJ, et al. The axonal attractant Netrin-1 is an angiogenic factor. Proc Natl Acad Sci U S A. 2004;101(46):16210–5. doi: 10.1073/pnas.0405984101 15520390 PMC528958

[48] van Gils JM, Derby MC, Fernandes LR, Ramkhelawon B, Ray TD, Rayner KJ, et al. The neuroimmune guidance cue netrin-1 promotes atherosclerosis by inhibiting the emigration of macrophages from plaques. Nat Immunol. 2012;13(2):136–43. doi: 10.1038/ni.2205 22231519 PMC3262880

[49] Ramkhelawon B, Hennessy EJ, Ménager M, Ray TD, Sheedy FJ, Hutchison S, et al. Netrin-1 promotes adipose tissue macrophage retention and insulin resistance in obesity. Nat Med. 2014;20(4):377–84. doi: 10.1038/nm.3467 24584118 PMC3981930

[50] Hardy H, Prendergast JG, Patel A, Dutta S, Trejo-Reveles V, Kroeger H. Detailed analysis of chick optic fissure closure reveals Netrin-1 as an essential mediator of epithelial fusion. eLife. 2019;8:e43877. doi: 10.7554/eLife.43877 PMC6606025 31162046

[51] Howe LJ, Richardson TG, Arathimos R, Alvizi L, Passos-Bueno MR, Stanier P, et al. Evidence for DNA methylation mediating genetic liability to non-syndromic cleft lip/palate. Epigenomics. 2019;11(2):133–45. doi: 10.2217/epi-2018-0091 30638414 PMC6462847

[52] Hampl M, Jandová N, Lusková D, Nováková M, Szotkowská T, Čada Š, et al. Early embryogenesis in CHDFIDD mouse model reveals facial clefts and altered cranial neurogenesis. Dis Model Mech. 2024;17(6):dmm050261. doi: 10.1242/dmm.050261 38511331 PMC11212636

[53] Guo H, Bettella E, Marcogliese PC, Zhao R, Andrews JC, Nowakowski TJ, et al. Disruptive mutations in TANC2 define a neurodevelopmental syndrome associated with psychiatric disorders. Nat Commun. 2019;10(1):4679. doi: 10.1038/s41467-019-12435-8 31616000 PMC6794285

[54] Tassano E, Accogli A, Ronchetto P, Tortora D, Tavella E, Gimelli G, et al. 17q23.3 de novo microdeletion involving only TANC2 gene: A new case. Eur J Med Genet. 2020;63(12):104094. doi: 10.1016/j.ejmg.2020.104094 33160097

[55] Wessel K, Suleiman J, Khalaf TE, Kishore S, Rolfs A, El-Hattab AW. 17q23.2q23.3 de novo duplication in association with speech and language disorder, learning difficulties, incoordination, motor skill impairment, and behavioral disturbances: a case report. BMC Med Genet. 2017;18(1):119. 29070031 10.1186/s12881-017-0479-3 PMC5657100

[56] Yang Q, Liu H, Li Z, Wang Y, Liu W. Purification and mutagenesis studies of TANC1 ankyrin repeats domain provide clues to understand mis-sense variants from diseases. Biochem Biophys Res Commun. 2019;514(2):358–64. doi: 10.1016/j.bbrc.2019.04.151 31040020

[57] Long F, Zheng J, Zhou J, Hu P, Xiong B. Knockout of tanc2 causes autism-like behavior and sleep disturbance in zebrafish. Autism Res. 2023;16(3):524–34. doi: 10.1002/aur.2880 36534563

[58] Leslie EJ, Liu H, Carlson JC, Shaffer JR, Feingold E, Wehby G, et al. A genome-wide association study of nonsyndromic cleft palate identifies an etiologic missense variant in GRHL3. Am J Hum Genet. 2016;98(4):744–54. 27018472 10.1016/j.ajhg.2016.02.014 PMC4833215

[59] Beaty TH, Murray JC, Marazita ML, Munger RG, Ruczinski I, Hetmanski JB, et al. A genome-wide association study of cleft lip with and without cleft palate identifies risk variants near MAFB and ABCA4. Nat Genet. 2010;42(6):525–9. doi: 10.1038/ng.580 20436469 PMC2941216

[60] Laurie CC, Doheny KF, Mirel DB, Pugh EW, Bierut LJ, Bhangale T, et al. Quality control and quality assurance in genotypic data for genome-wide association studies. Genet Epidemiol. 2010;34(6):591–602. doi: 10.1002/gepi.20516 20718045 PMC3061487

[61] Taliun D, Harris DN, Kessler MD, Carlson J, Szpiech ZA, Torres R, et al. Sequencing of 53,831 diverse genomes from the NHLBI TOPMed Program. Nature. 2021;590(7845):290–9. doi: 10.1038/s41586-021-03205-y 33568819 PMC7875770

[62] Chang CC, Chow CC, Tellier LC, Vattikuti S, Purcell SM, Lee JJ. Second-generation PLINK: rising to the challenge of larger and richer datasets. GigaScience. 2015;4(1):s13742-015-0047–8. 10.1186/s13742-015-0047-8 PMC4342193 25722852

[63] Gogarten SM, Sofer T, Chen H, Yu C, Brody JA, Thornton TA, et al. Genetic association testing using the GENESIS R/Bioconductor package. Bioinformatics. 2019;35(24):5346–8. doi: 10.1093/bioinformatics/btz567 31329242 PMC7904076

[64] Conomos MP, Gogarten SM, Brown L, Chen H, Rice K, Sofer T. GENESIS: GENetic EStimation and Inference in Structured samples (GENESIS): Statistical methods for analyzing genetic data from samples with population structure and/or relatedness. 2019. https://github.com/UW-GAC/GENESIS

[65] Watanabe K, Taskesen E, van Bochoven A, Posthuma D. Functional mapping and annotation of genetic associations with FUMA. Nat Commun. 2017;8(1):1826. 29184056 10.1038/s41467-017-01261-5 PMC5705698

[66] Barbeira AN, Dickinson SP, Bonazzola R, Zheng J, Wheeler HE, Torres JM. Exploring the phenotypic consequences of tissue specific gene expression variation inferred from GWAS summary statistics. Nature Communications. 2018;9(1):1825. doi: 10.1038/s41467-018-04256-1 PMC5940825 29739930

[67] Barbeira AN, Melia OJ, Liang Y, Bonazzola R, Wang G, Wheeler HE. Fine-mapping and QTL tissue-sharing information improves the reliability of causal gene identification. Genet Epidemiol. 2020;44(8):854–67. 32964524 10.1002/gepi.22346 PMC7693040

[68] Barbeira AN, Pividori M, Zheng J, Wheeler HE, Nicolae DL, Im HK. Integrating predicted transcriptome from multiple tissues improves association detection. PLoS Genet. 2019;15(1):e1007889. doi: 10.1371/journal.pgen.1007889 30668570 PMC6358100

