# Supplementary material for: Variants in CALD1, ESRP1, and RBFOX1 are associated with orofacial cleft risk
Source: PLoS Genet. 2025 Sep 30;21(9):e1011581. doi: 10.1371/journal.pgen.1011581 (PMC12503292; doi:10.1371/journal.pgen.1011581)
Supplement: S1 Text — (DOCX) [file pgen.1011581.s012.docx]

**Fig A.** **Manhattan and quantile-quantile plots of meta-analysis results from METAL for any cleft (ANY).** Plots were created with LocusZoom[1]. Genomic control factor based on the median, λ_GC_, was 1.028.


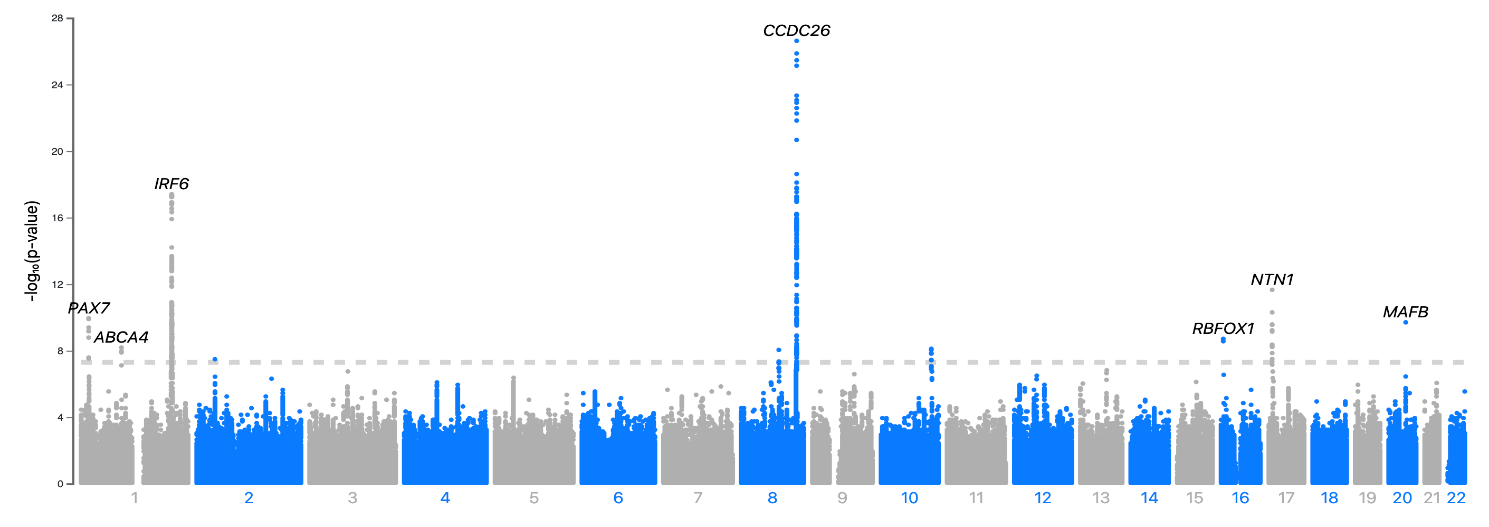

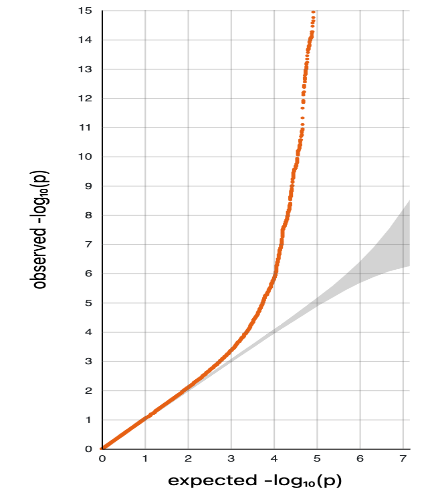


**Fig B.** **Manhattan and quantile-quantile plots of meta-analysis results from METAL for cleft lip with/without cleft palate (CL/P).** Plots were created with LocusZoom [2]. Genomic control factor based on the median, λ_GC_, was 1.024.


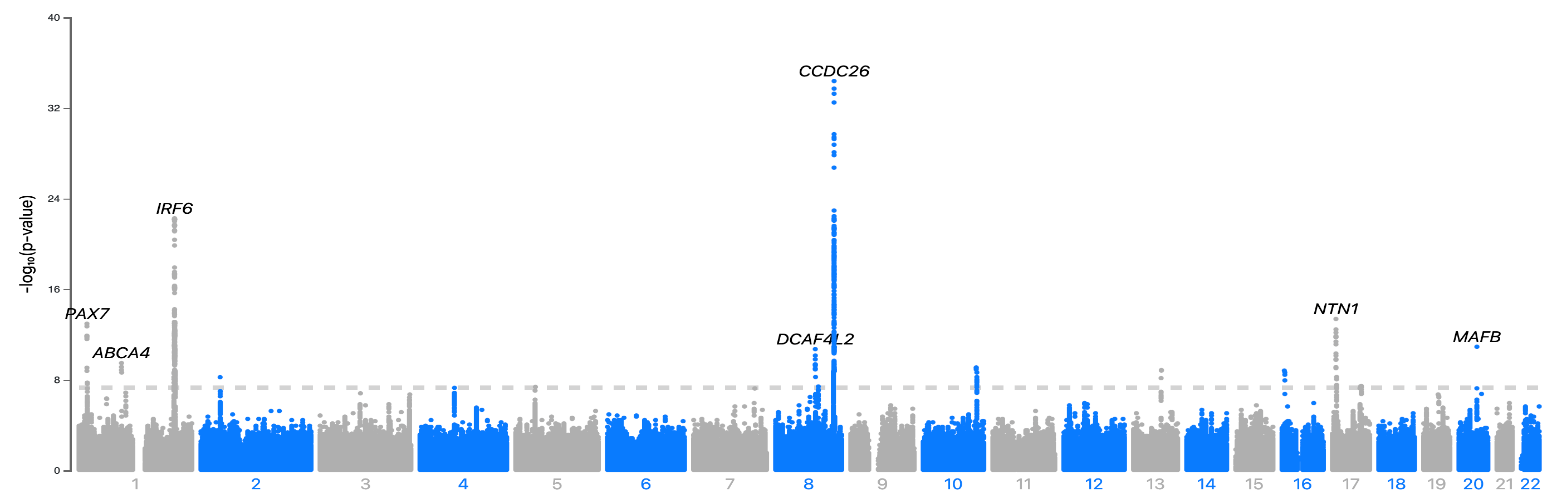

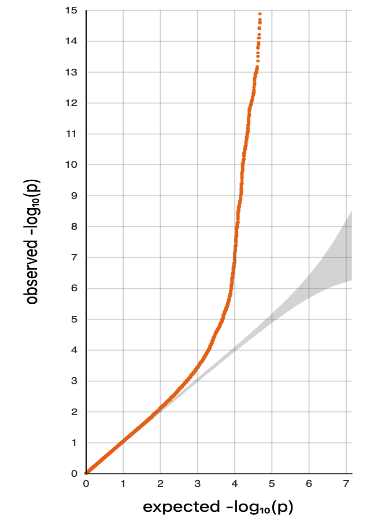


**Fig C.** **Manhattan and quantile-quantile plots of meta-analysis results from METAL for cleft lip and palate (CLP).** Plots were created with LocusZoom [2]. Genomic control factor based on the median, λ_GC_, was 1.02.


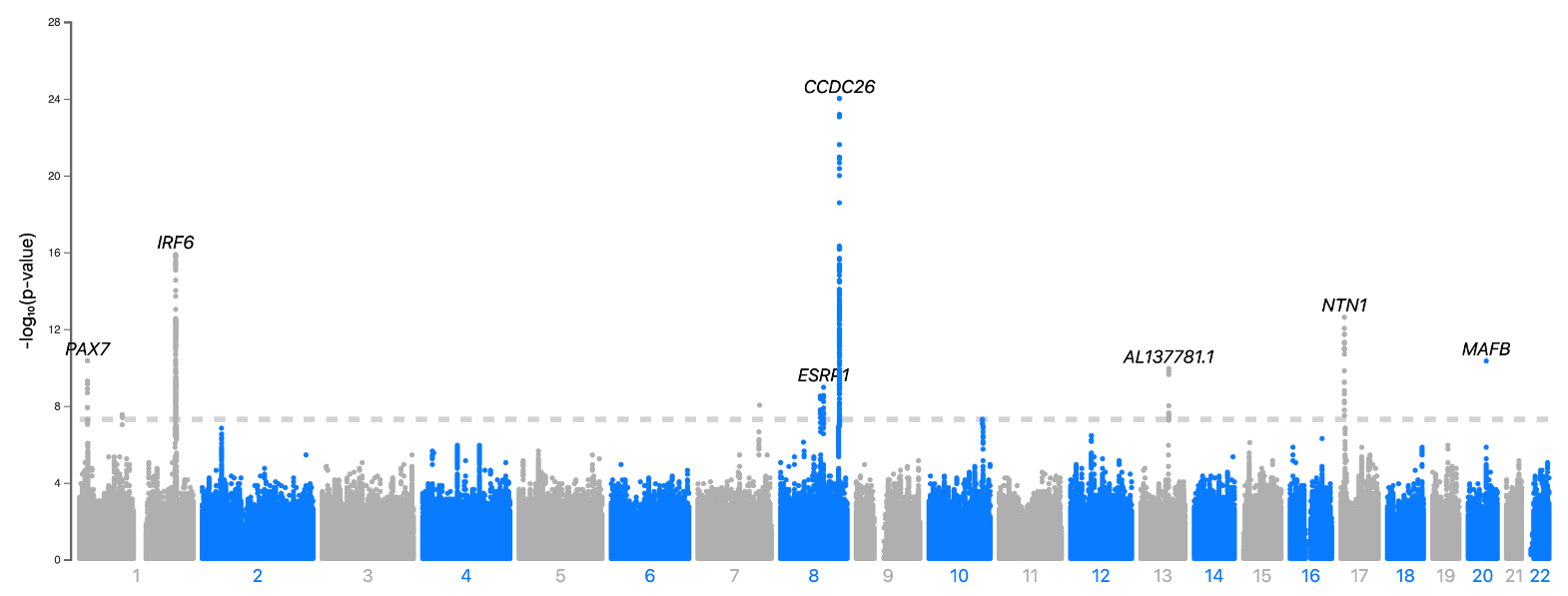

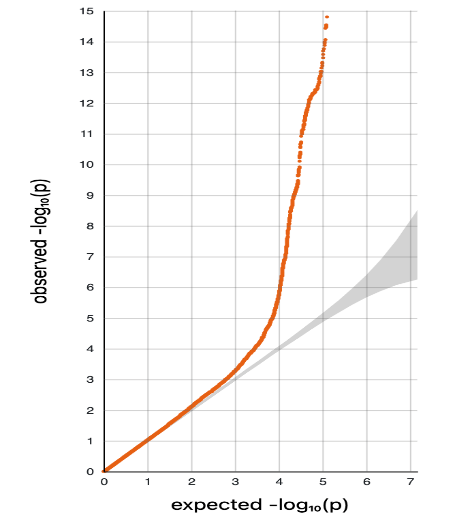


**Fig D.** **Manhattan and quantile-quantile plots of meta-analysis results from METAL for cleft lip only (CLO).** Plots were created with LocusZoom [2]. Genomic control factor based on the median, λ_GC_, was 0.99.


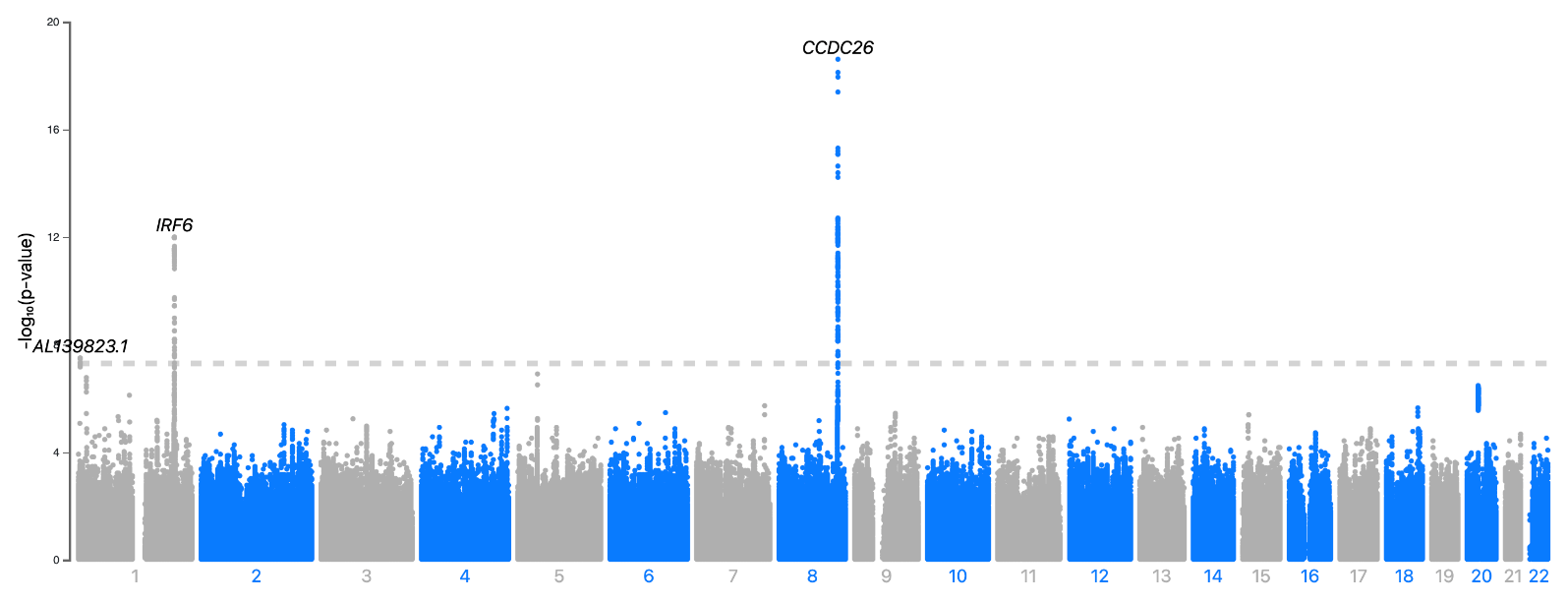

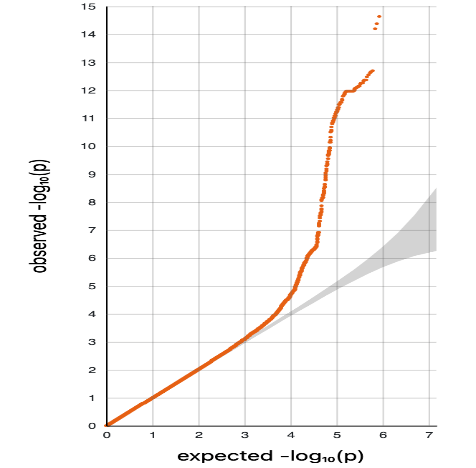


**Fig E.** **Manhattan and quantile-quantile plots of meta-analysis results from METAL for cleft palate only (CPO).** Plots were created with LocusZoom [2]. Genomic control factor based on the median, λ_GC_, was 1.00.


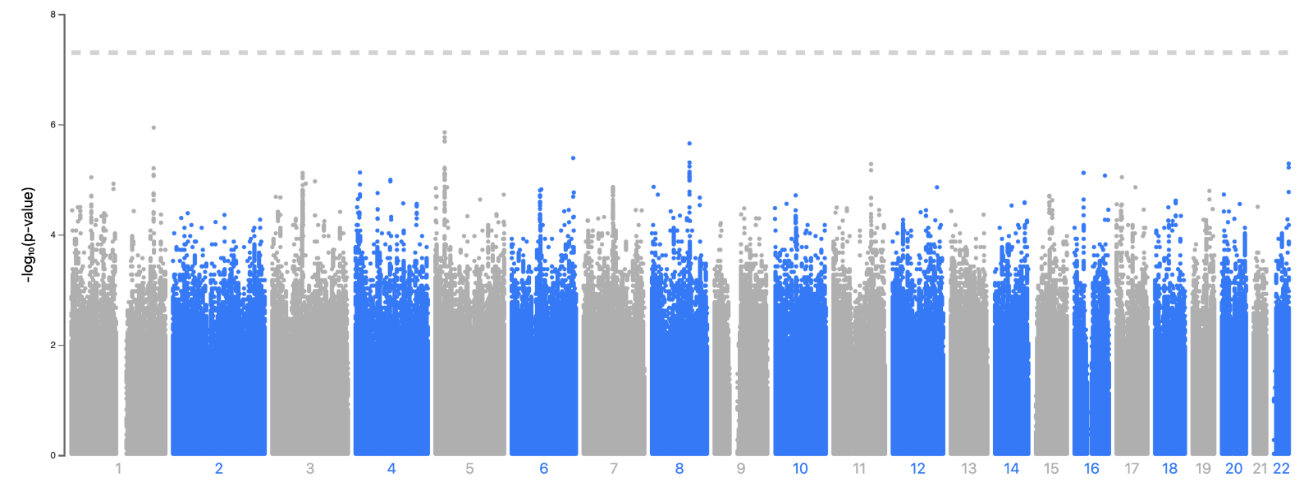

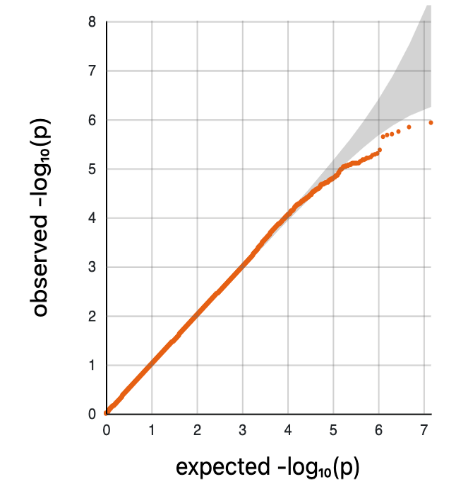

**Fig F. Manhattan and quantile-quantile plots of multi-tissue TWAS results from S‑MulTiXcan for any cleft (ANY).** Plots were created with LocusZoom [2] and qqman [3]. Genomic control factor based on the median, λ_GC_, was 0.92.


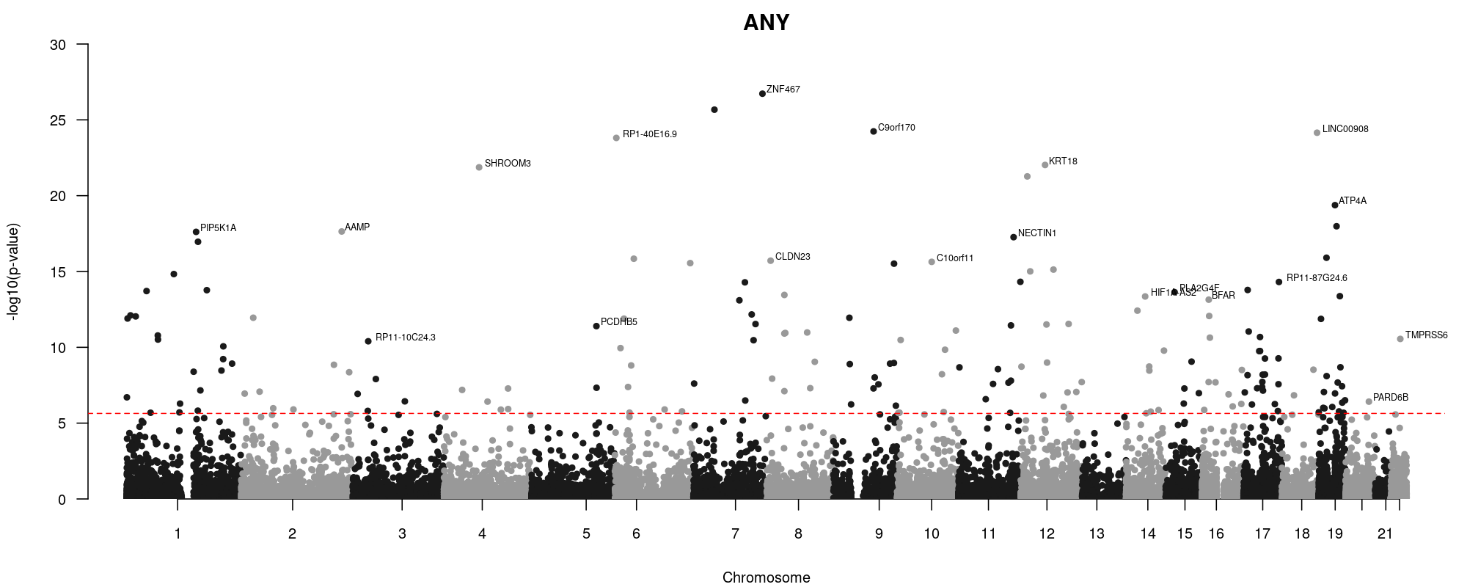

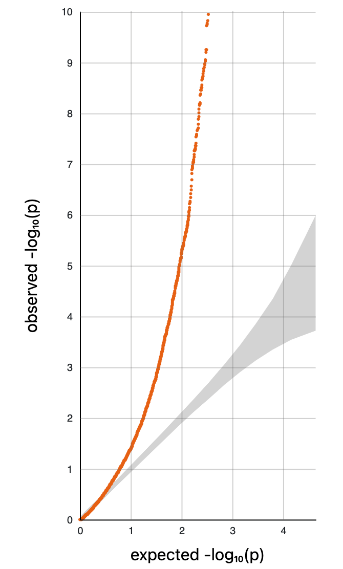


**Fig G. Manhattan and quantile-quantile plots of multi-tissue TWAS results from S‑MulTiXcan cleft lip with/without cleft palate (CL/P).** Plots were created with LocusZoom [2] and qqman [3]. Genomic control factor based on the median, λ_GC_, was 0.941.


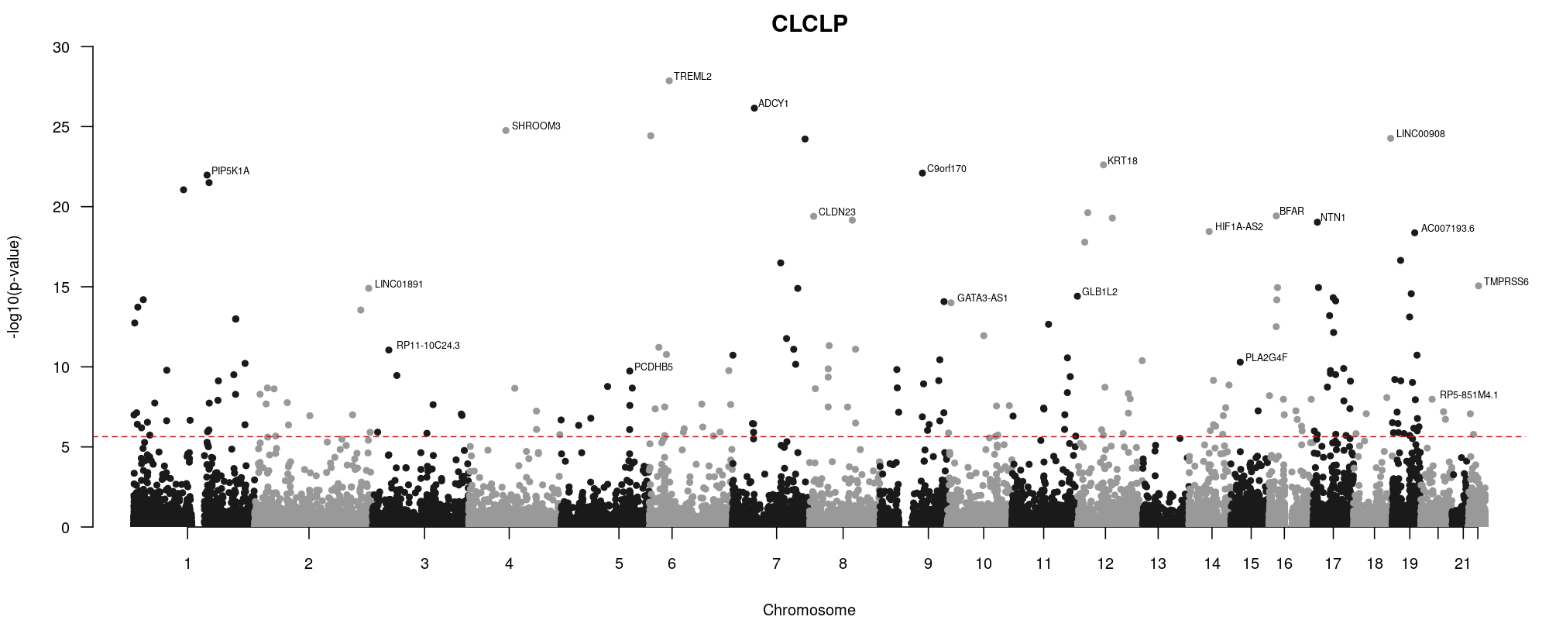

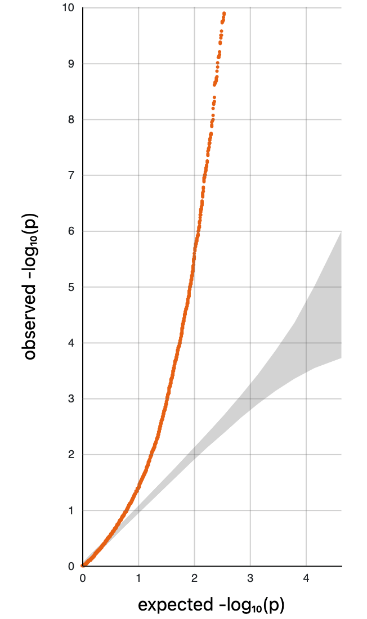


**Fig H. Manhattan and quantile-quantile plots of multi-tissue TWAS results from S‑MulTiXcan for cleft lip and palate (CLP).** Plots were created with LocusZoom [2] and qqman [3]. Genomic control factor based on the median, λ_GC_, was 0.917.


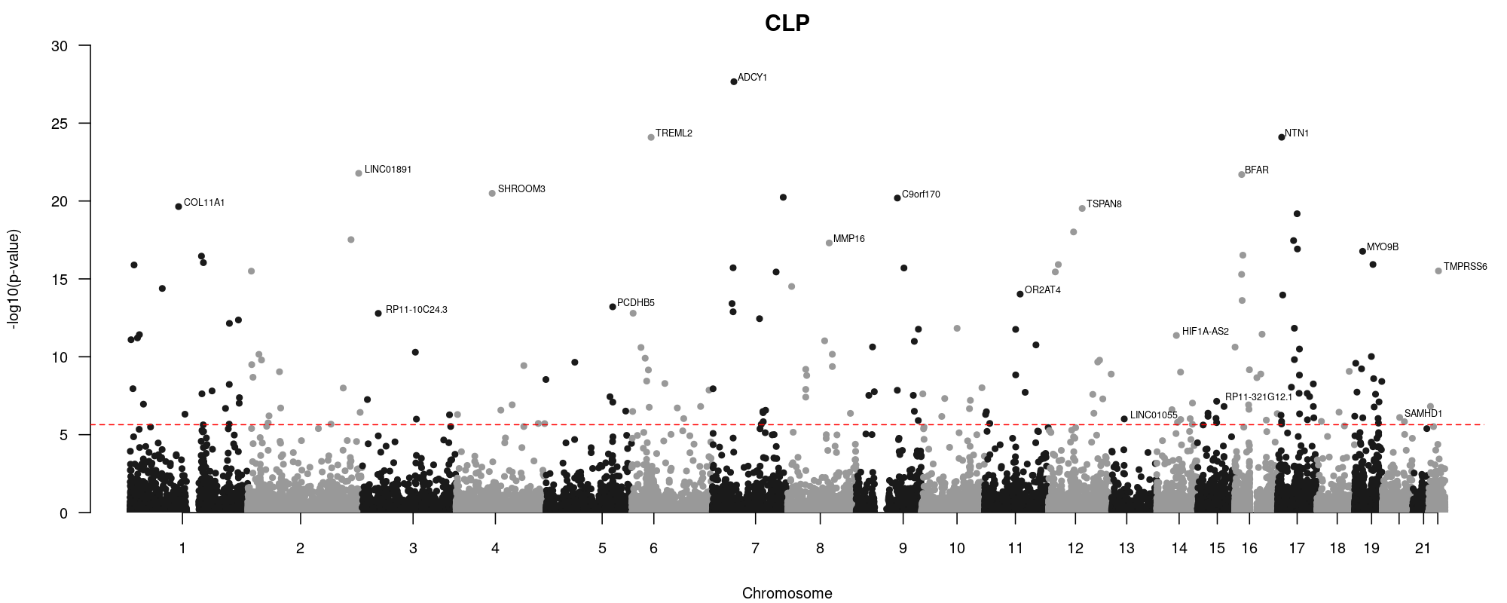

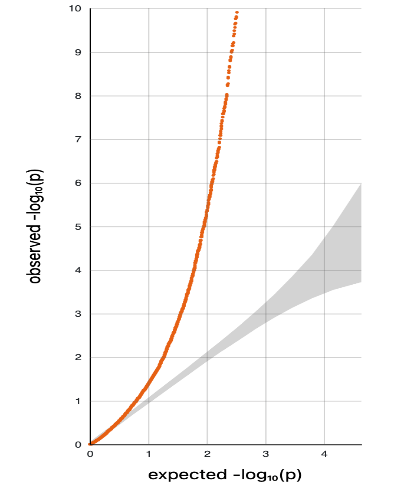


**Fig I. Manhattan and quantile-quantile plots of multi-tissue TWAS results from S‑MulTiXcan for cleft lip only (CLO).** Plots were created with LocusZoom [2] and qqman [3]. Genomic control factor based on the median, λ_GC_, was 0.842.


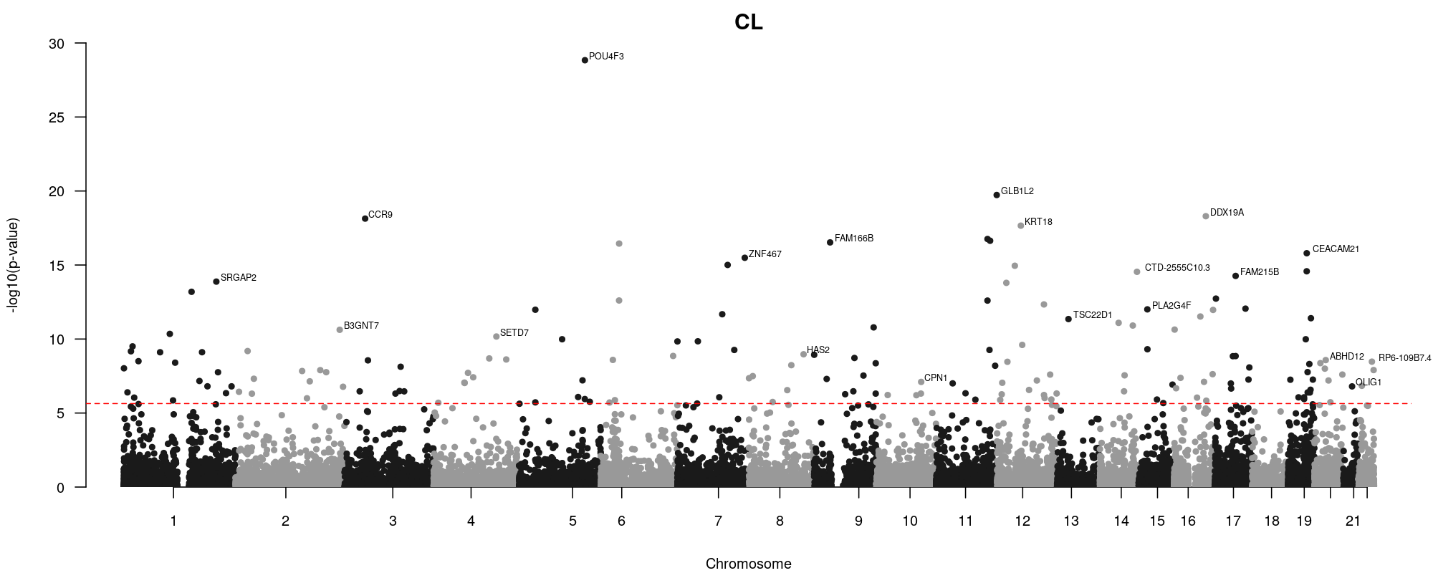

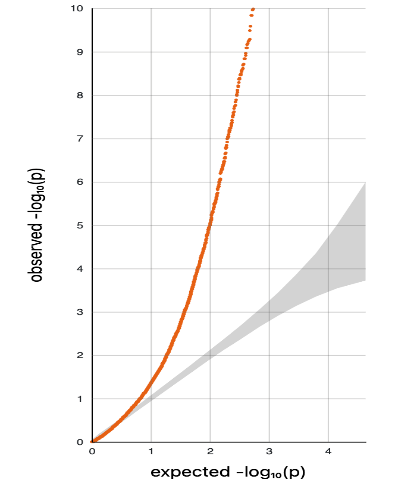


**Fig J. Manhattan and quantile-quantile plots of multi-tissue TWAS results from S‑MulTiXcan for cleft palate only (CPO).** Plots were created with LocusZoom [2] and qqman [3]. Genomic control factor based on the median, λ_GC_, was 0.856.


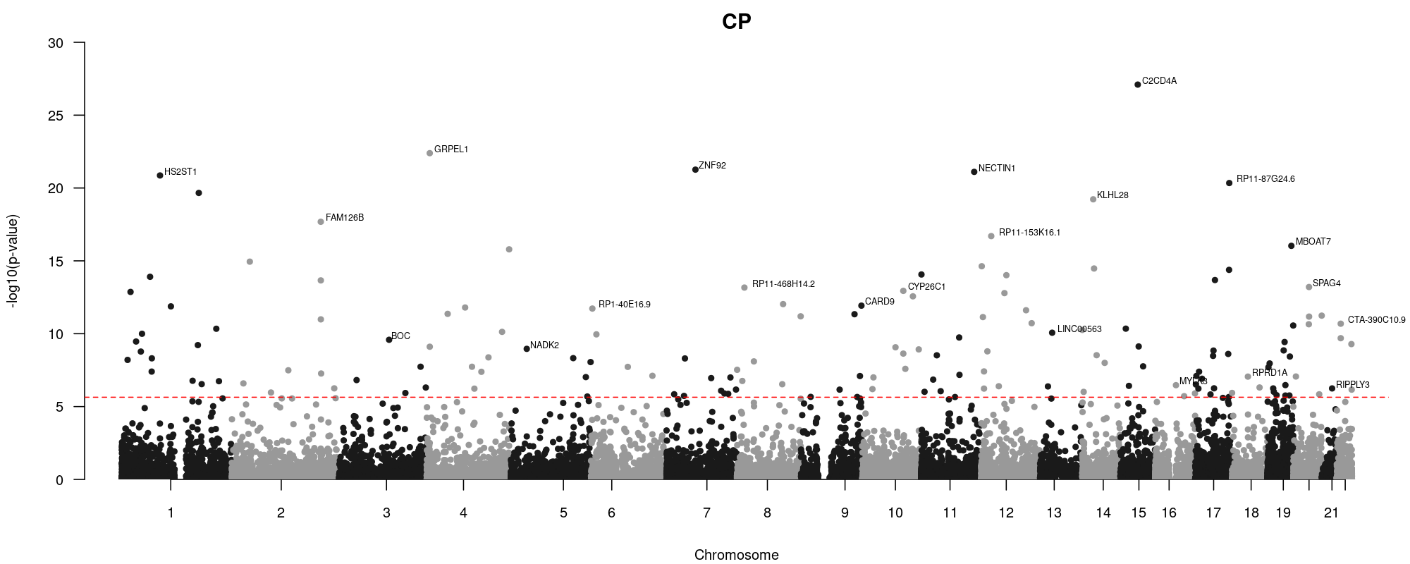

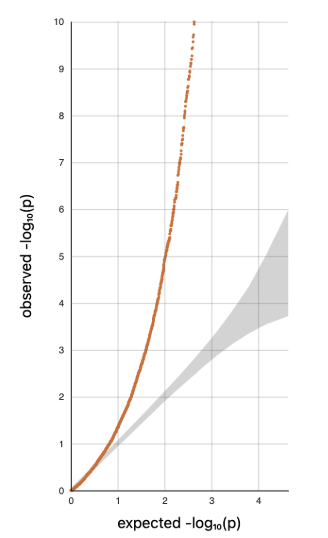


**Fig K.** **Manhattan and quantile-quantile plots of the Asian ancestry meta-analysis results from METAL for any cleft (ANY).** Plots were created with LocusZoom [2]. Genomic control factor based on the median, λ_GC_, was 1.001.


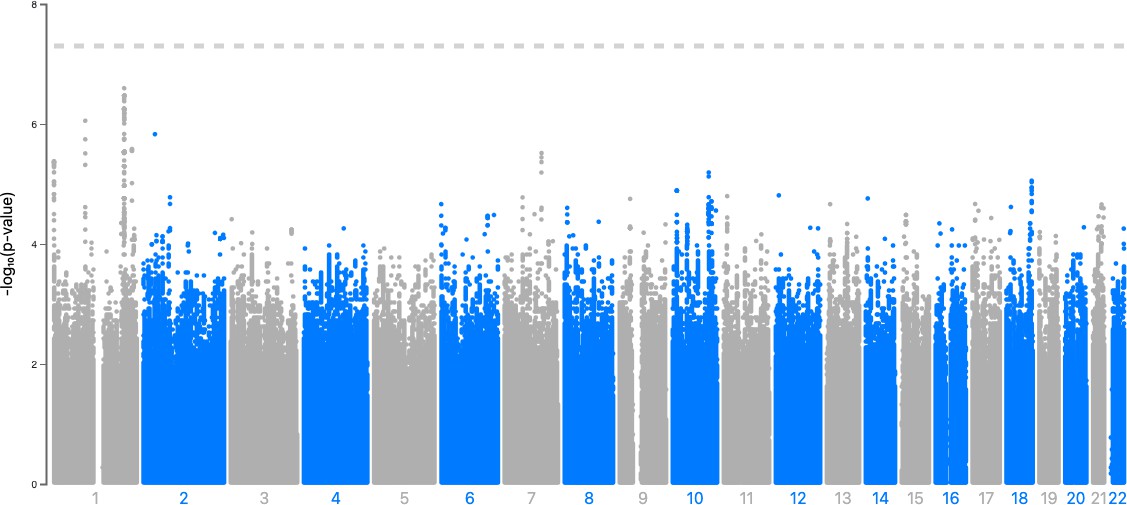

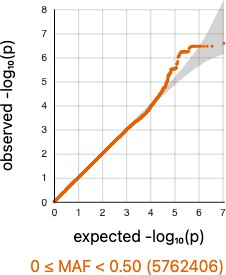


**Fig L. Manhattan and quantile-quantile plots of the Asian ancestry meta-analysis results from METAL for cleft lip with/without cleft palate (CL/P).** Plots were created with LocusZoom [2]. Genomic control factor based on the median, λ_GC_, was 1.005.


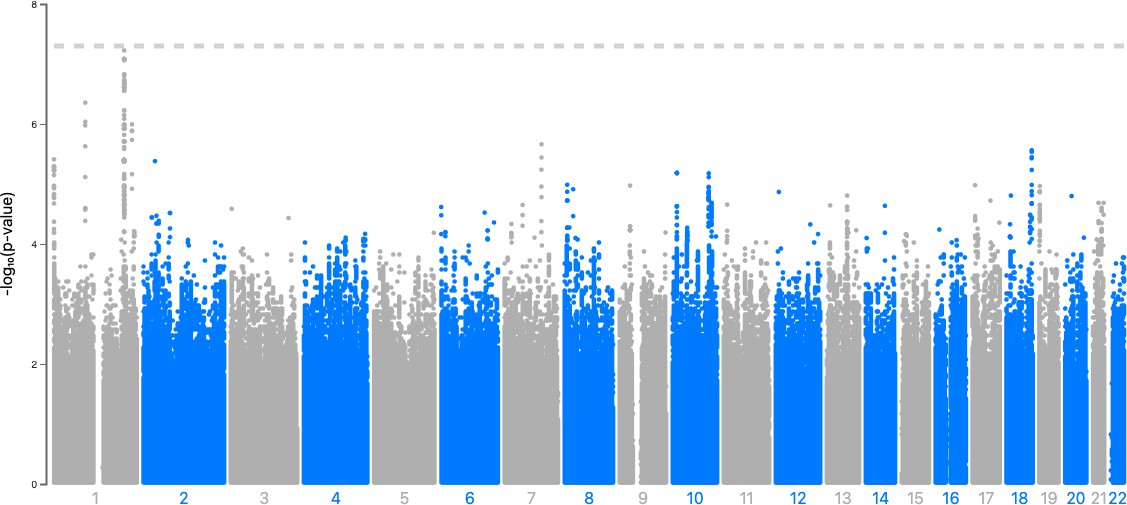

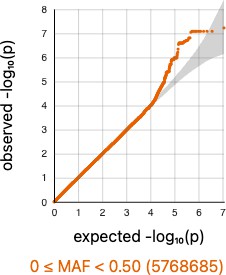


**Fig M.** **Manhattan and quantile-quantile plots of the Asian ancestry meta-analysis results from METAL for cleft lip and palate (CLP).** Plots were created with LocusZoom [2]. Genomic control factor based on the median, λ_GC_, was 1.008.


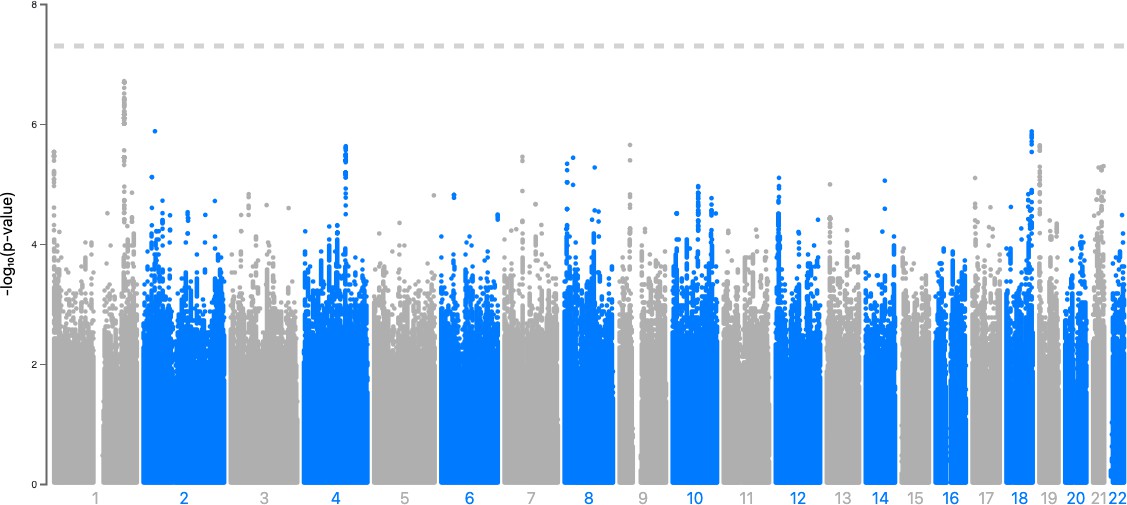

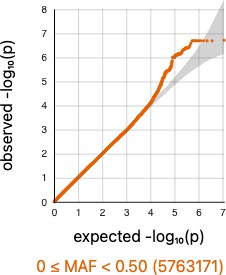


**Fig N.** **Manhattan and quantile-quantile plots of the Asian ancestry meta-analysis results from METAL for cleft lip only (CLO).** Plots were created with LocusZoom [2]. Genomic control factor based on the median, λ_GC_, was 0.997.


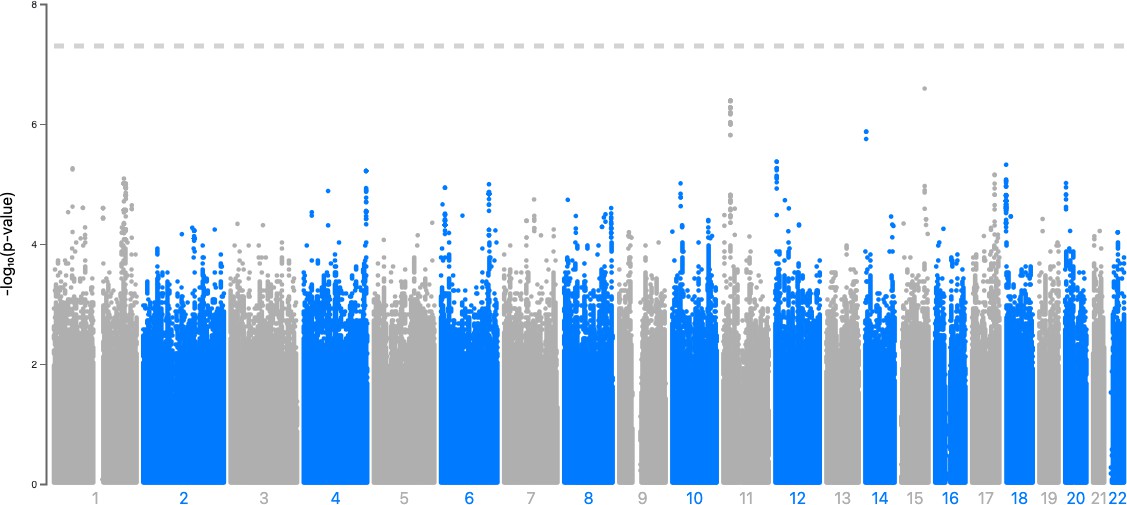

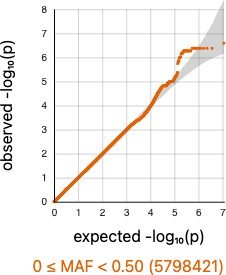


**Fig O.** **Manhattan and quantile-quantile plots of the Asian ancestry meta-analysis results from METAL for cleft palate only (CPO).** Plots were created with LocusZoom [2]. Genomic control factor based on the median, λ_GC_, was 0.983.


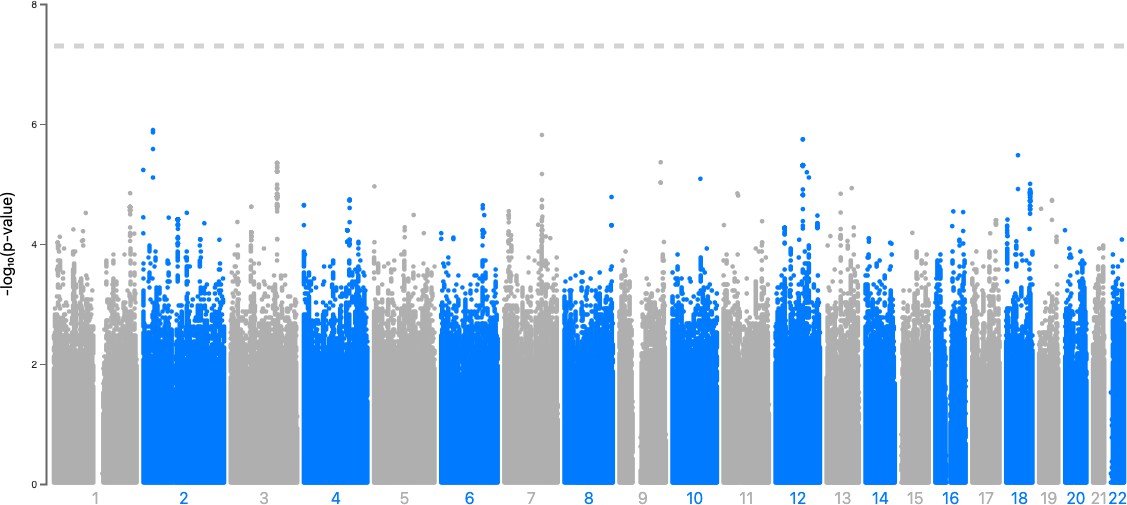

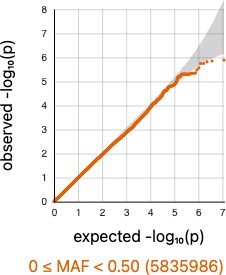


**Fig P. Manhattan and quantile-quantile plots of the Asian ancestry multi-tissue TWAS results from S‑MulTiXcan for any cleft (ANY).** Plots were created with LocusZoom [2] and qqman [3]. Genomic control factor based on the median, λ_GC_, was 1.002.


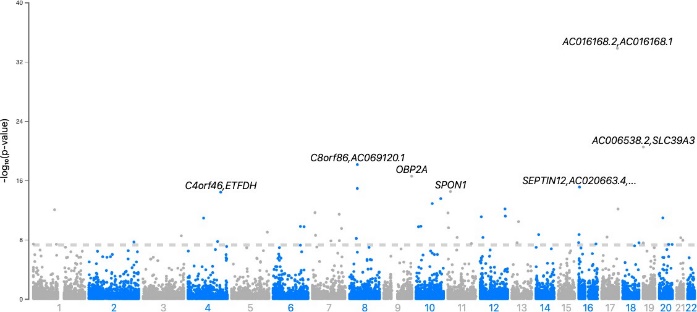

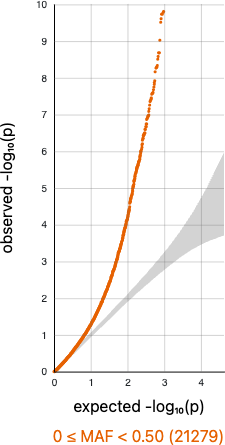


**Fig Q. Manhattan and quantile-quantile plots of the Asian ancestry multi-tissue TWAS results from S‑MulTiXcan cleft lip with/without cleft palate (CL/P).** Plots were created with LocusZoom [2] and qqman [3]. Genomic control factor based on the median, λ_GC_, was 1.013.


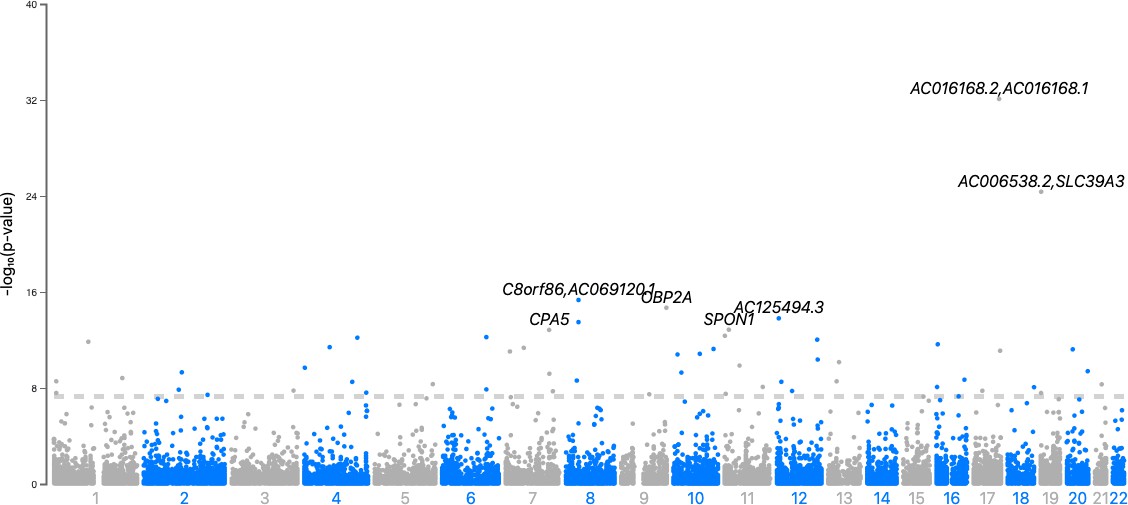

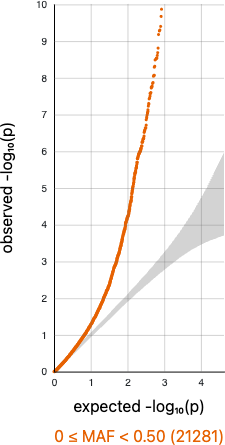


**Fig R. Manhattan and quantile-quantile plots of the Asian ancestry multi-tissue TWAS results from S‑MulTiXcan for cleft lip and palate (CLP).** Plots were created with LocusZoom [2] and qqman [3]. Genomic control factor based on the median, λ_GC_, was 0.988.


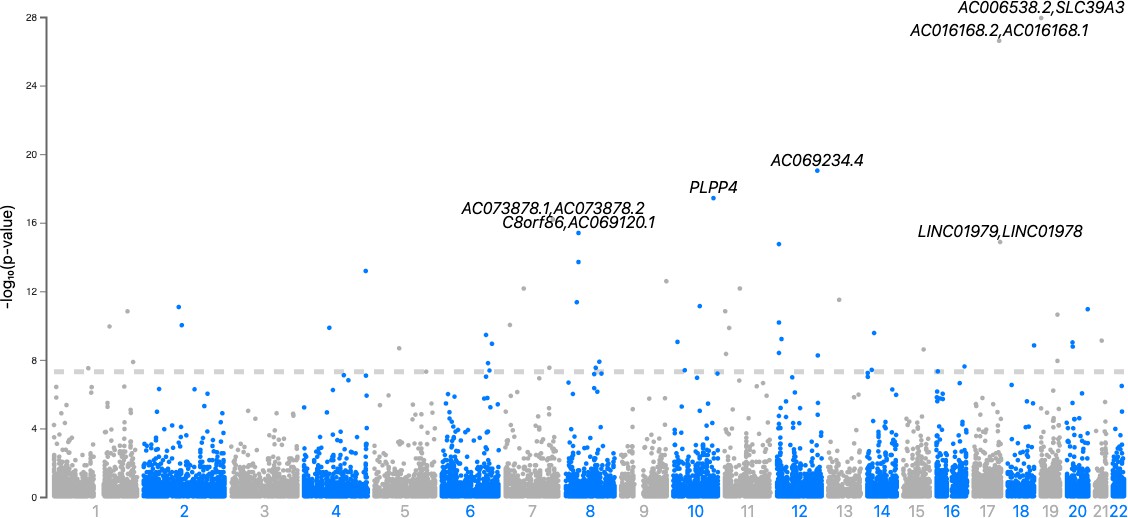

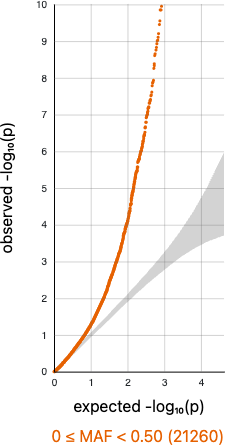


**Fig S. Manhattan and quantile-quantile plots of the Asian ancestry multi-tissue TWAS results from S‑MulTiXcan for cleft lip only (CLO).** Plots were created with LocusZoom [2] and qqman [3]. Genomic control factor based on the median, λ_GC_, was 0.971.


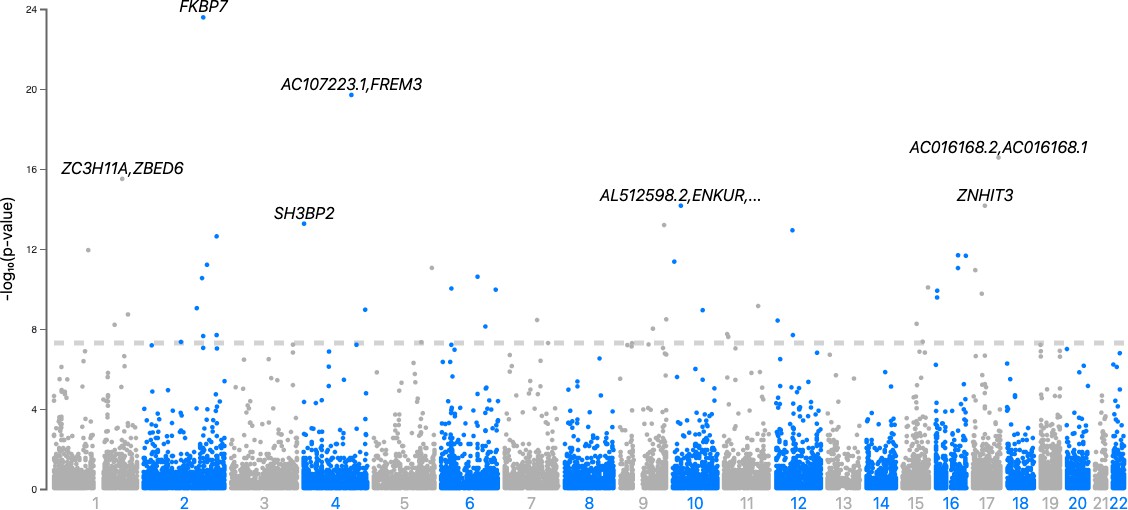

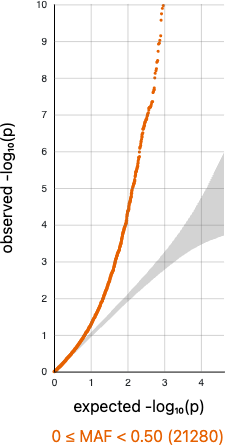


**Fig T. Manhattan and quantile-quantile plots of the Asian ancestry multi-tissue TWAS results from S‑MulTiXcan for cleft palate only (CPO).** Plots were created with LocusZoom [2] and qqman [3]. Genomic control factor based on the median, λ_GC_, was 0.942.


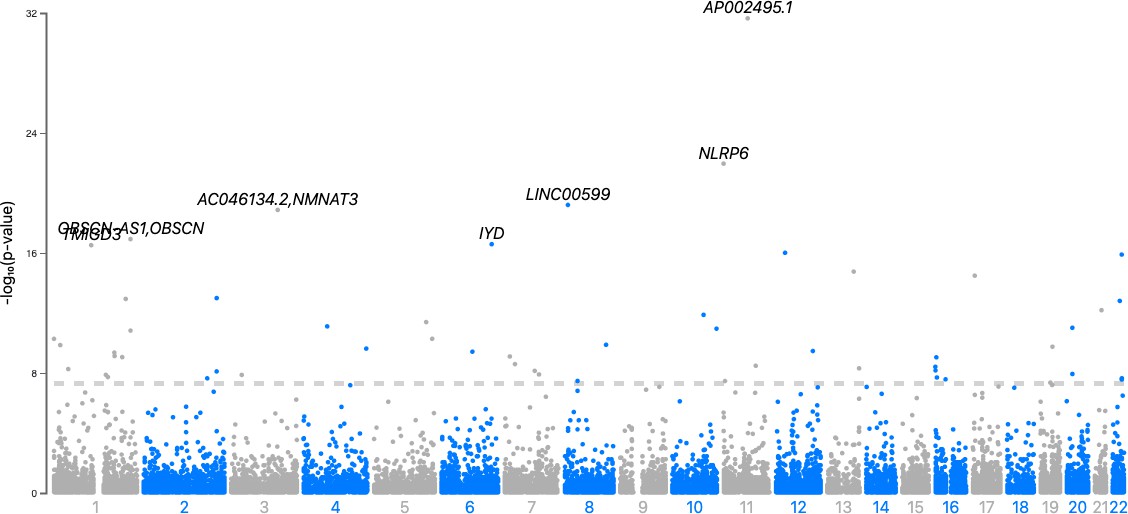

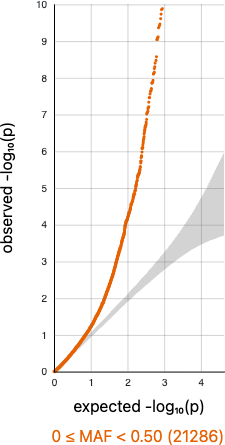


**Fig U.** **Manhattan and quantile-quantile plots of the European ancestry meta-analysis results from METAL for any cleft (ANY).** Plots were created with LocusZoom [2]. Genomic control factor based on the median, λ_GC_, was 1.012.


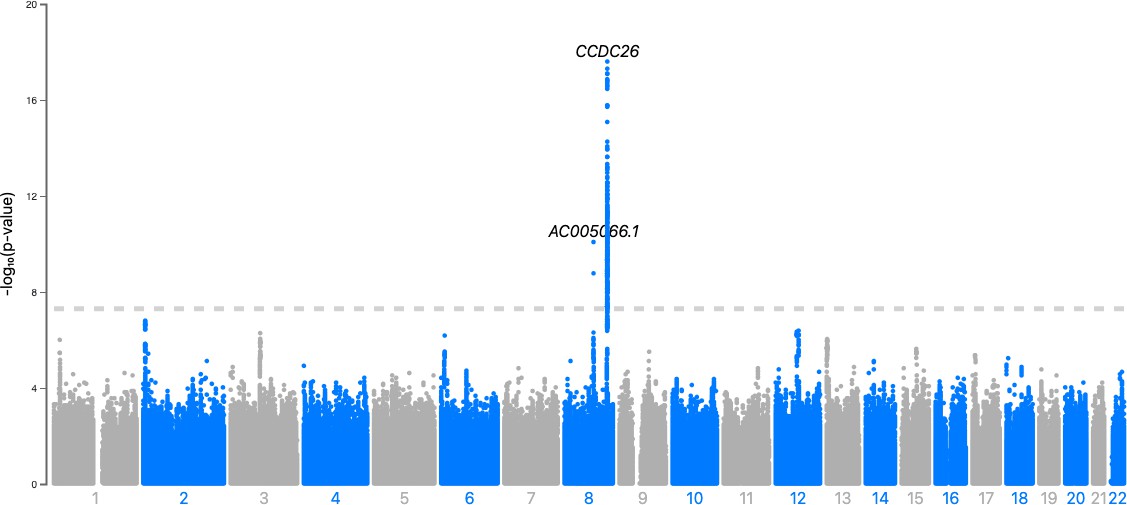

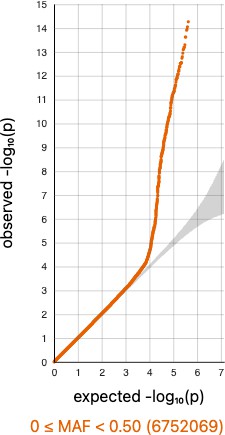


**Fig V. Manhattan and quantile-quantile plots of the European ancestry meta-analysis results from METAL for cleft lip with/without cleft palate (CL/P).** Plots were created with LocusZoom [2]. Genomic control factor based on the median, λ_GC_, was 1.009.


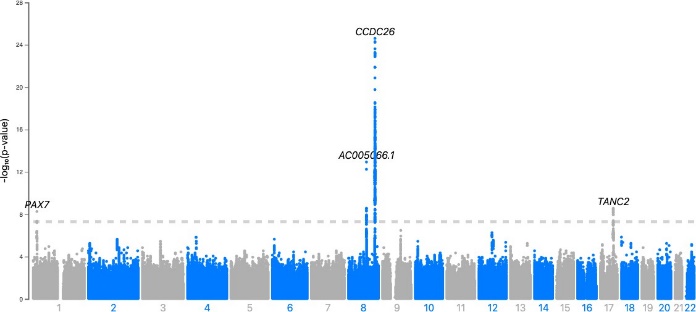

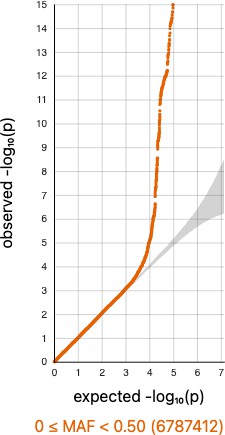


**Fig W.** **Manhattan and quantile-quantile plots of the European ancestry meta-analysis results from METAL for cleft lip and palate (CLP).** Plots were created with LocusZoom [2]. Genomic control factor based on the median, λ_GC_, was 1.008.


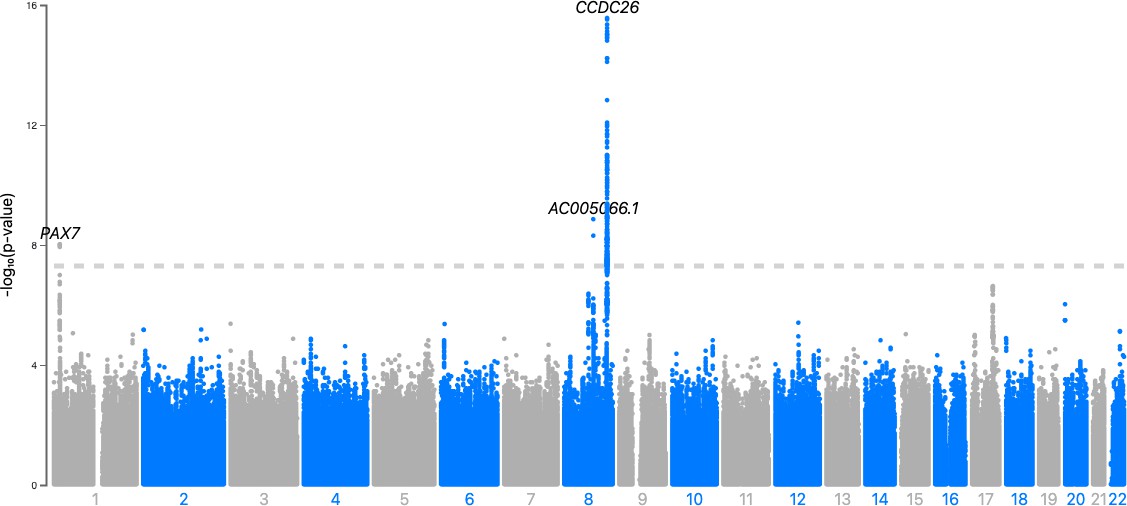

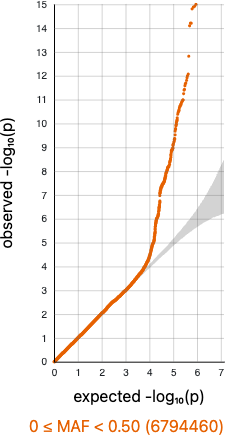


**Fig X.** **Manhattan and quantile-quantile plots of the European ancestry meta-analysis results from METAL for cleft lip only (CLO).** Plots were created with LocusZoom [2]. Genomic control factor based on the median, λ_GC_, was 1.012.


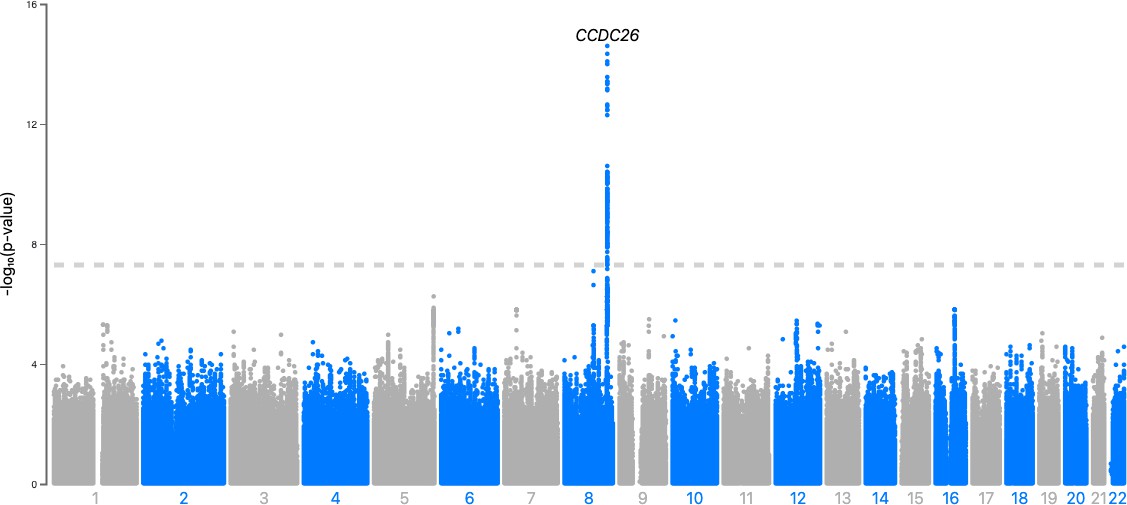

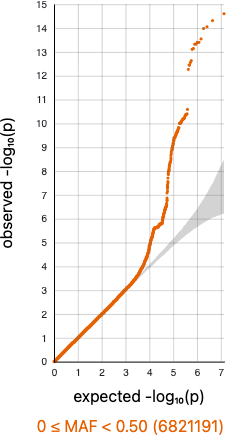


**Fig Y.** **Manhattan and quantile-quantile plots of the European ancestry meta-analysis results from METAL for cleft palate only (CPO).** Plots were created with LocusZoom [2]. Genomic control factor based on the median, λ_GC_, was 1.005.


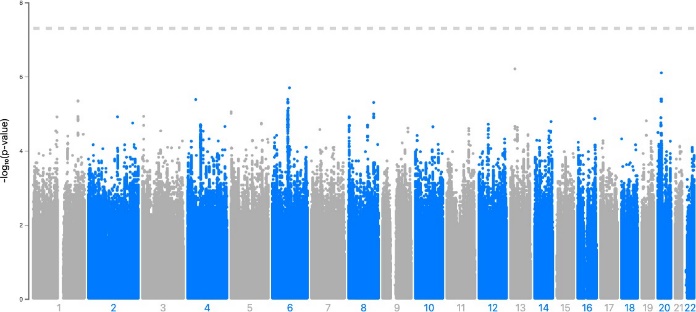

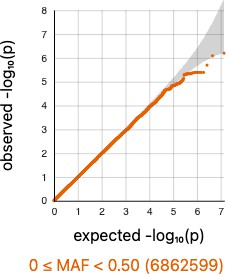


**Fig Z. Manhattan and quantile-quantile plots of the European ancestry multi-tissue TWAS results from S‑MulTiXcan for any cleft (ANY).** Plots were created with LocusZoom [2] and qqman [3]. Genomic control factor based on the median, λ_GC_, was 1.124.


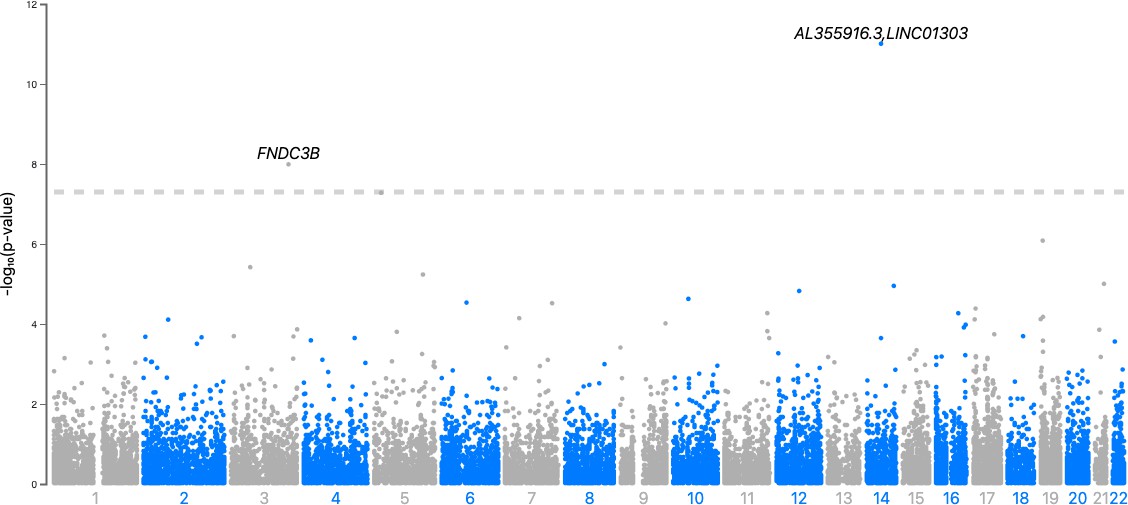

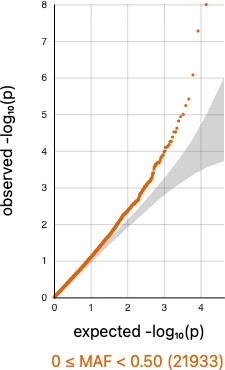


**Fig AA. Manhattan and quantile-quantile plots of the European ancestry multi-tissue TWAS results from S‑MulTiXcan cleft lip with/without cleft palate (CL/P).** Plots were created with LocusZoom [2] and qqman [3]. Genomic control factor based on the median, λ_GC_, was 1.129.


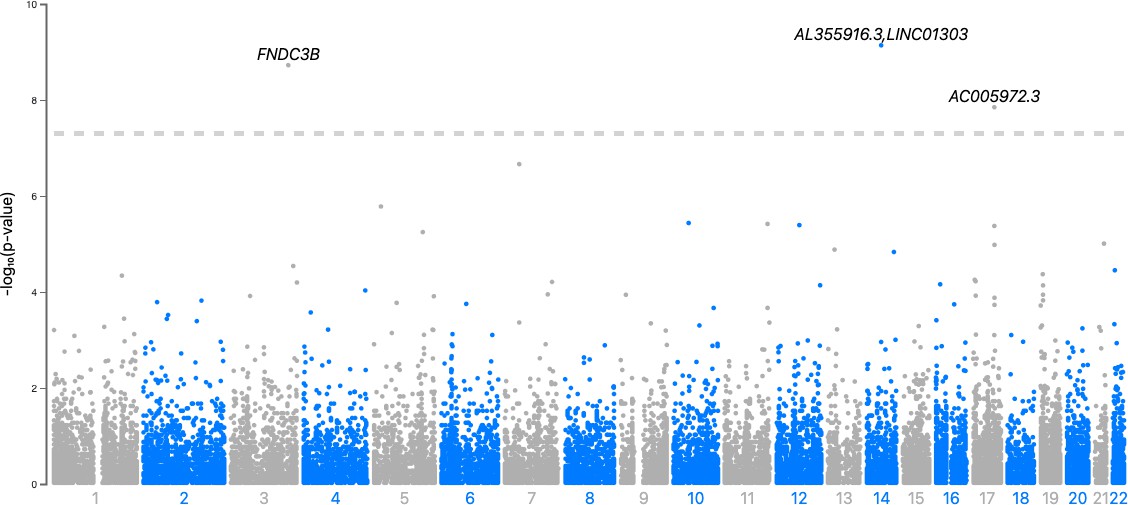

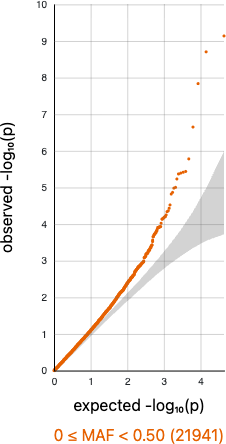


**Fig AB. Manhattan and quantile-quantile plots of the European ancestry multi-tissue TWAS results from S‑MulTiXcan for cleft lip and palate (CLP).** Plots were created with LocusZoom [2] and qqman [3]. Genomic control factor based on the median, λ_GC_, was 1.088.


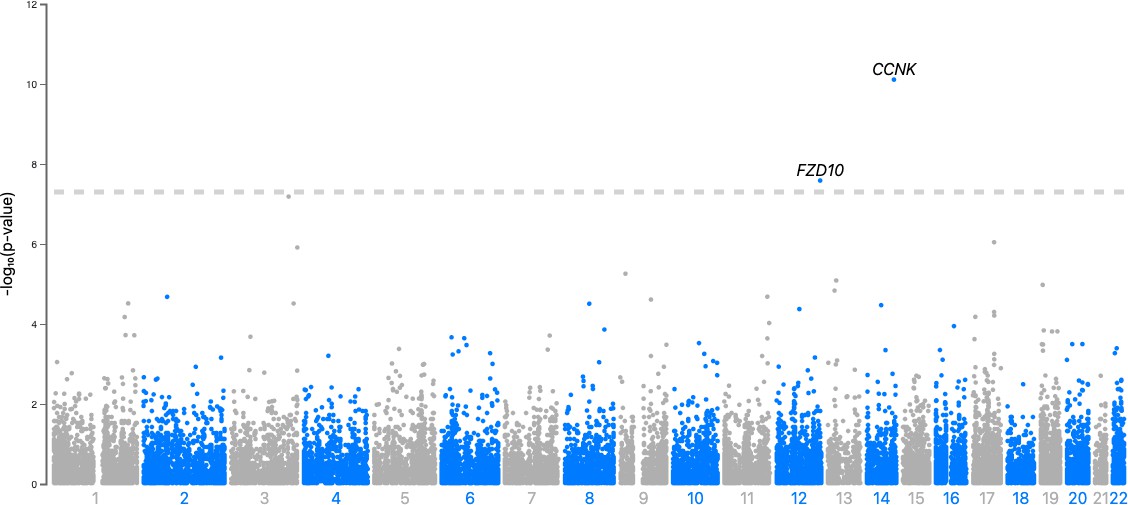

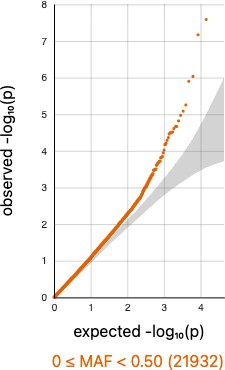


**Fig AC. Manhattan and quantile-quantile plots of the European ancestry multi-tissue TWAS results from S‑MulTiXcan for cleft lip only (CLO).** Plots were created with LocusZoom [2] and qqman [3]. Genomic control factor based on the median, λ_GC_, was 1.092.


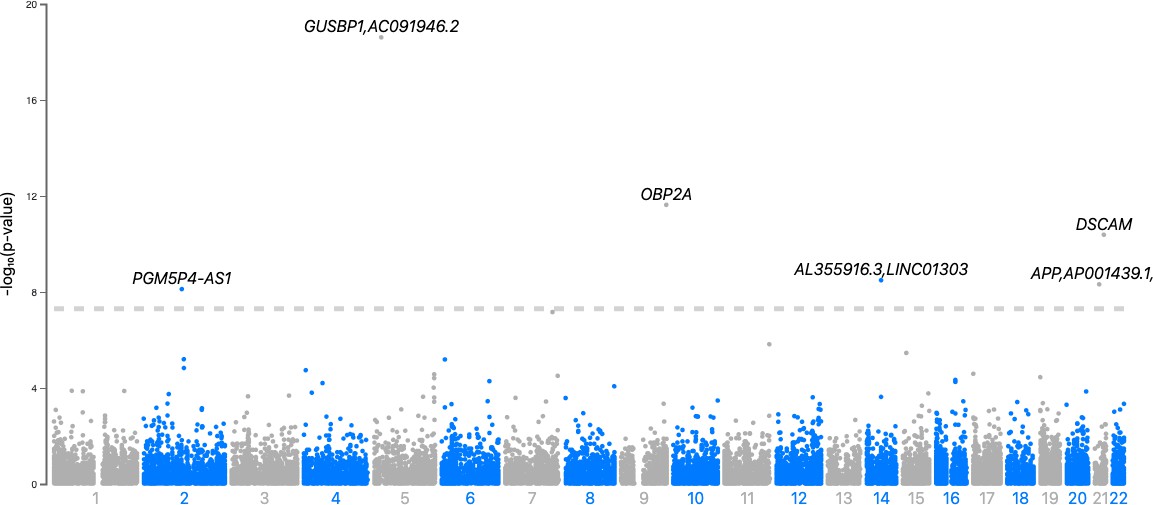

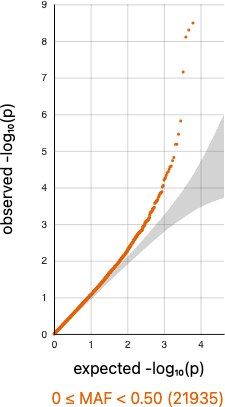


**Fig AD. Manhattan and quantile-quantile plots of the European ancestry multi-tissue TWAS results from S‑MulTiXcan for cleft palate only (CPO).** Plots were created with LocusZoom [2] and qqman [3]. Genomic control factor based on the median, λ_GC_, was 1.043.


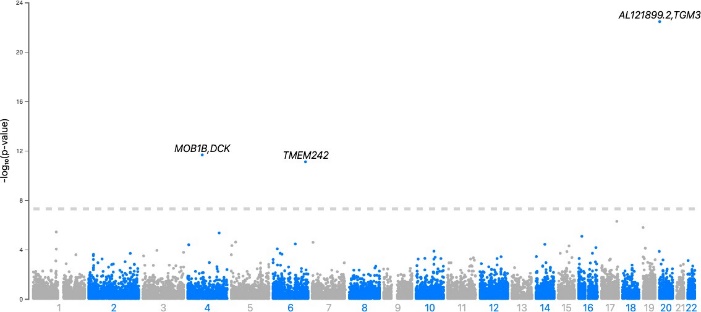

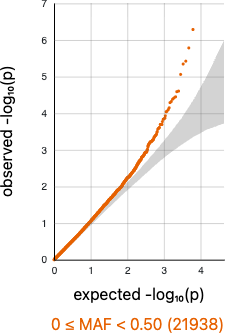


**Fig AE.** **Manhattan and quantile-quantile plots of the Central/South American ancestry meta-analysis results from METAL for any cleft (ANY).** Plots were created with LocusZoom [2]. Genomic control factor based on the median, λ_GC_, was 1.006.


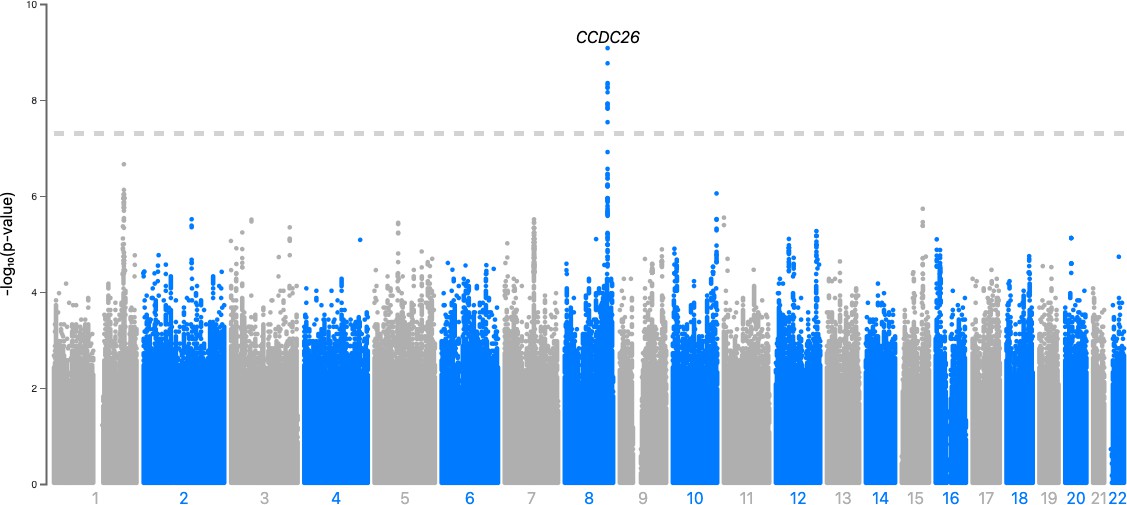

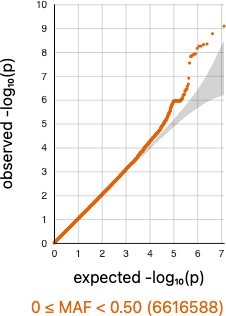


**Fig AF. Manhattan and quantile-quantile plots of the Central/South American ancestry meta-analysis results from METAL for cleft lip with/without cleft palate (CL/P).** Plots were created with LocusZoom [2]. Genomic control factor based on the median, λ_GC_, was 1.009.


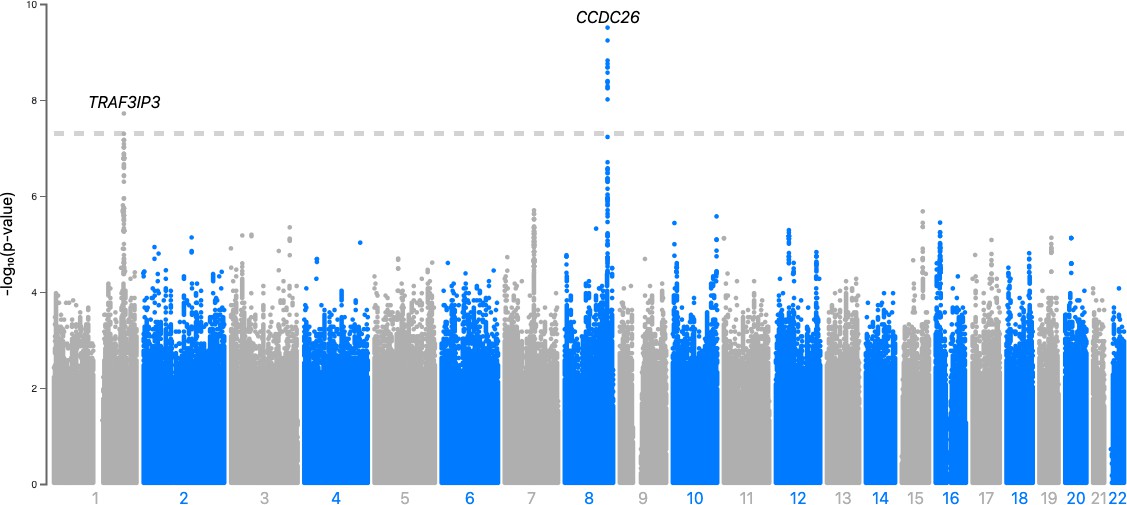

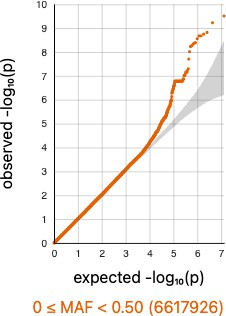


**Fig AG.** **Manhattan and quantile-quantile plots of the Central/South American ancestry meta-analysis results from METAL for cleft lip and palate (CLP).** Plots were created with LocusZoom [2]. Genomic control factor based on the median, λ_GC_, was 1.016.


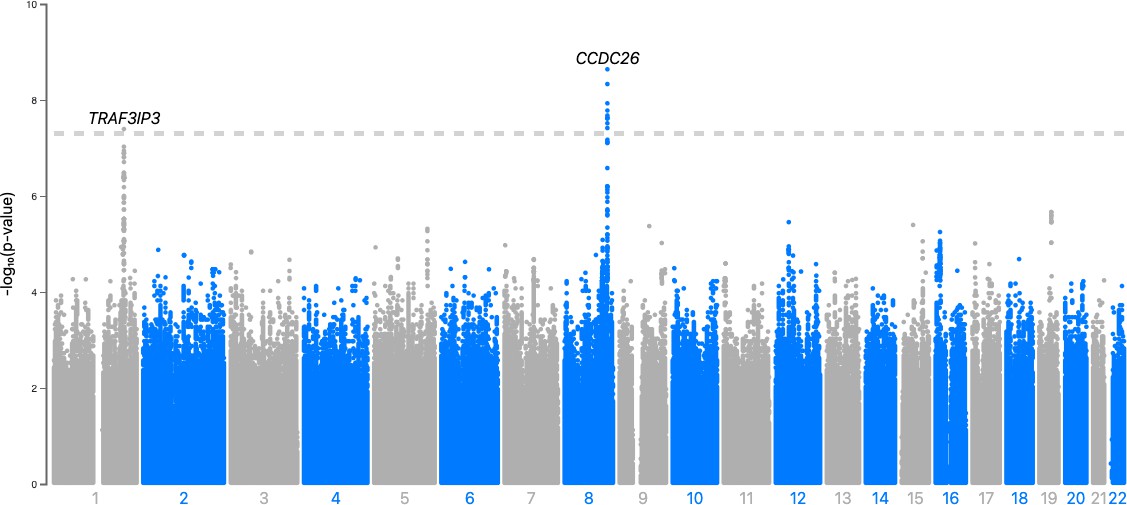

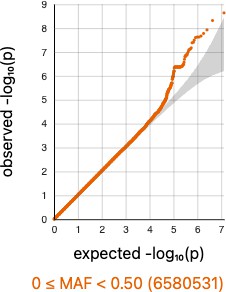


**Fig AH.** **Manhattan and quantile-quantile plots of the Central/South American ancestry meta-analysis results from METAL for cleft lip only (CLO).** Plots were created with LocusZoom [2]. Genomic control factor based on the median, λ_GC_, was 1.013.


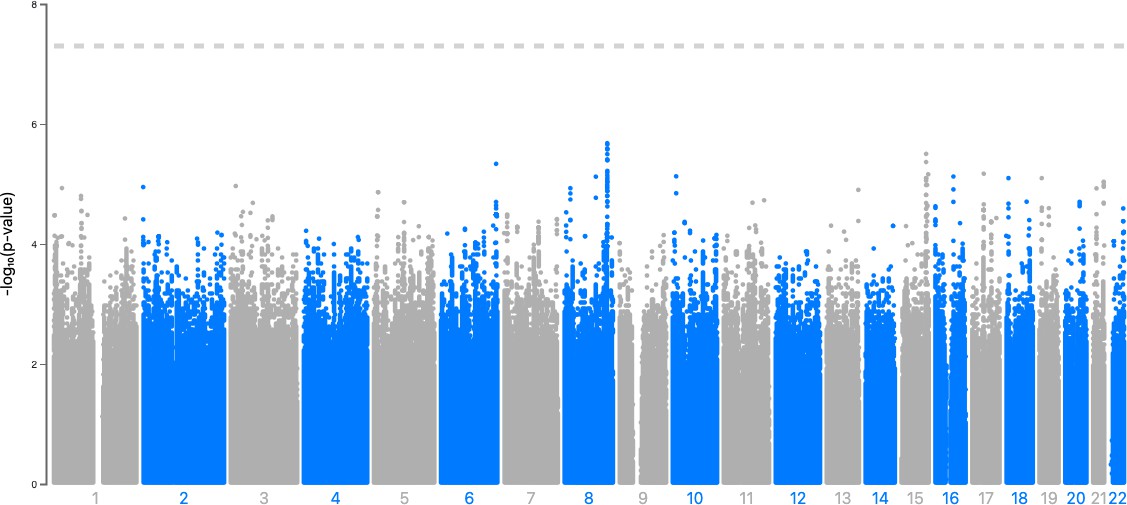

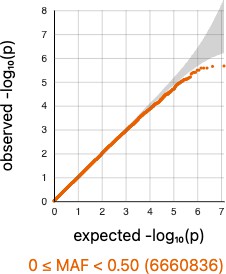


**Fig AI.** **Manhattan and quantile-quantile plots of the Central/South American ancestry meta-analysis results from METAL for cleft palate only (CPO).** Plots were created with LocusZoom [2]. Genomic control factor based on the median, λ_GC_, was 0.61.


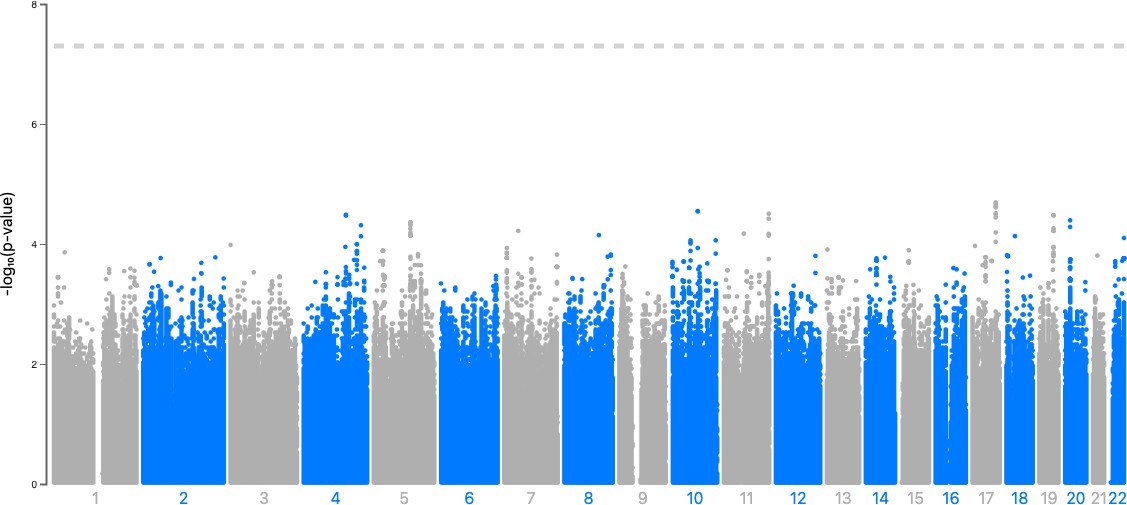

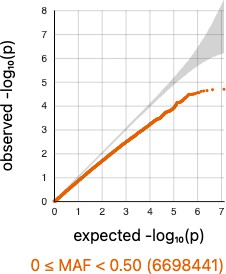


**Fig AJ. Manhattan and quantile-quantile plots of the Central/South American ancestry multi-tissue TWAS results from S‑MulTiXcan for any cleft (ANY).** Plots were created with LocusZoom [2] and qqman [3]. Genomic control factor based on the median, λ_GC_, was 1.085.


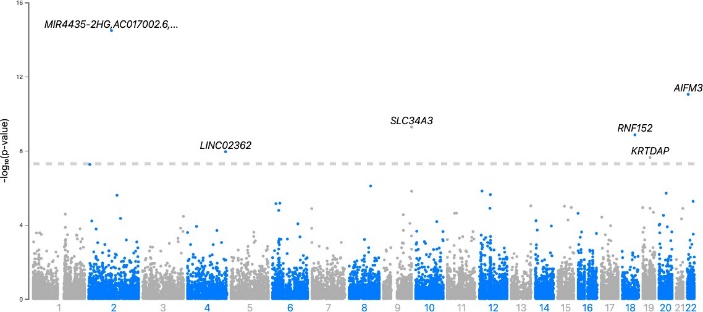

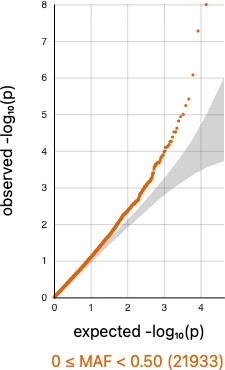


**Fig AK. Manhattan and quantile-quantile plots of the Central/South American ancestry multi-tissue TWAS results from S‑MulTiXcan cleft lip with/without cleft palate (CL/P).** Plots were created with LocusZoom [2] and qqman [3]. Genomic control factor based on the median, λ_GC_, was 1.072.


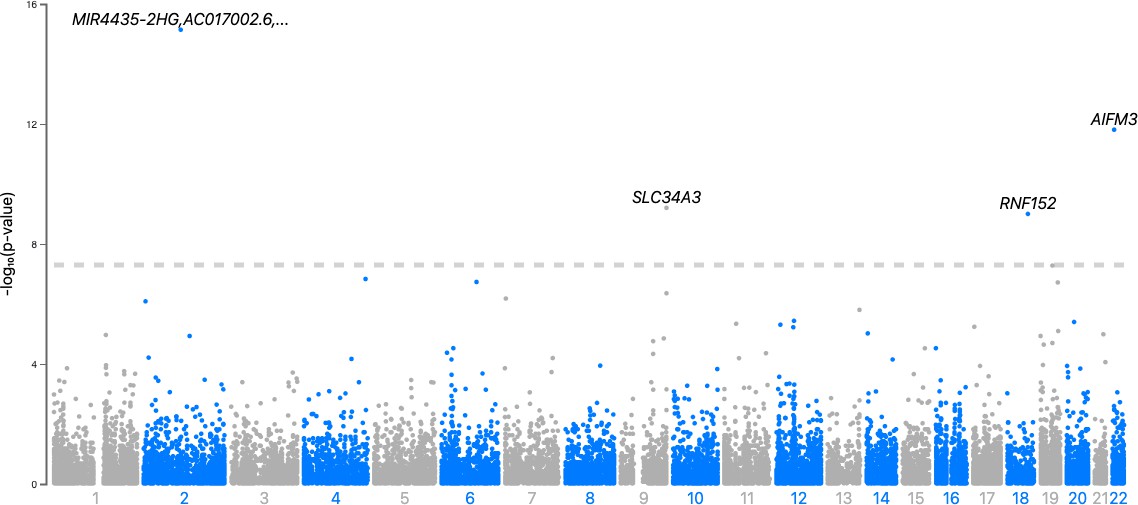

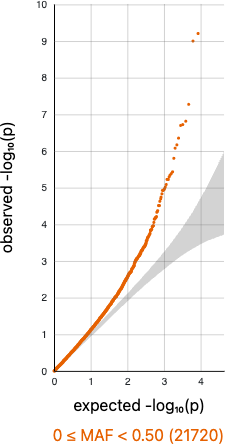


**Fig AL. Manhattan and quantile-quantile plots of the Central/South American ancestry multi-tissue TWAS results from S‑MulTiXcan for cleft lip and palate (CLP).** Plots were created with LocusZoom [2] and qqman [3]. Genomic control factor based on the median, λ_GC_, was 1.102.


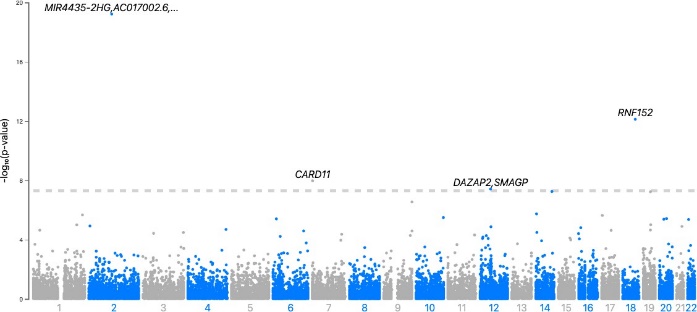

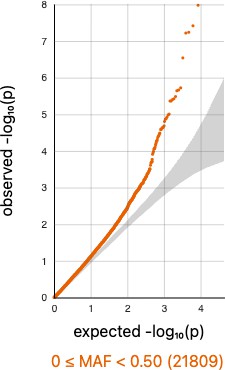


**Fig AM. Manhattan and quantile-quantile plots of the Central/South American ancestry multi-tissue TWAS results from S‑MulTiXcan for cleft lip only (CLO).** Plots were created with LocusZoom [2] and qqman [3]. Genomic control factor based on the median, λ_GC_, was 1.065.


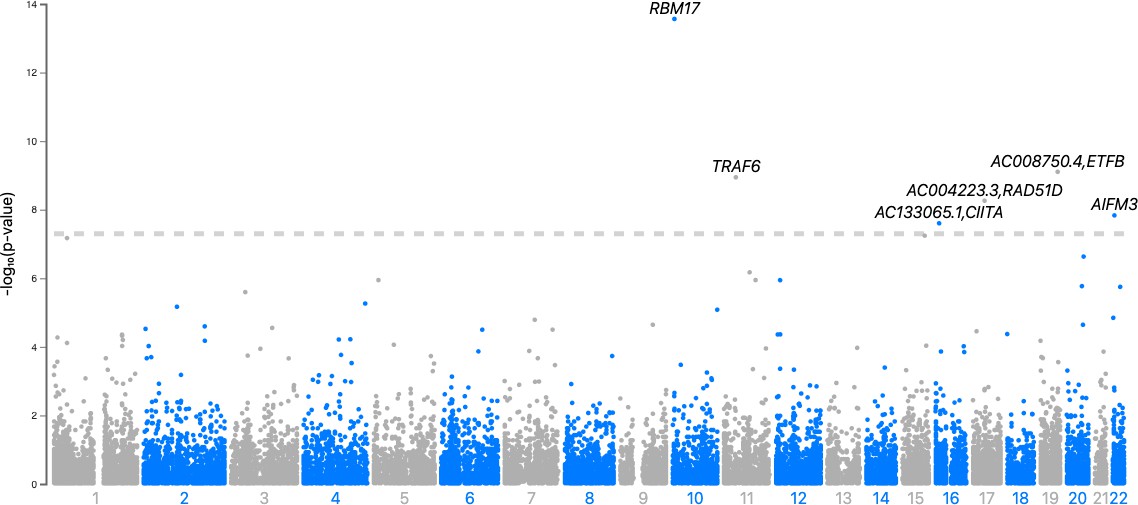

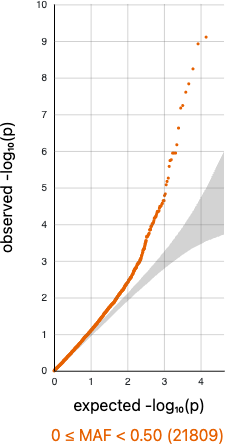


**Fig AN. Manhattan and quantile-quantile plots of the Central/South American ancestry multi-tissue TWAS results from S‑MulTiXcan for cleft palate only (CPO).** Plots were created with LocusZoom [2] and qqman [3]. Genomic control factor based on the median, λ_GC_, was 0.517.


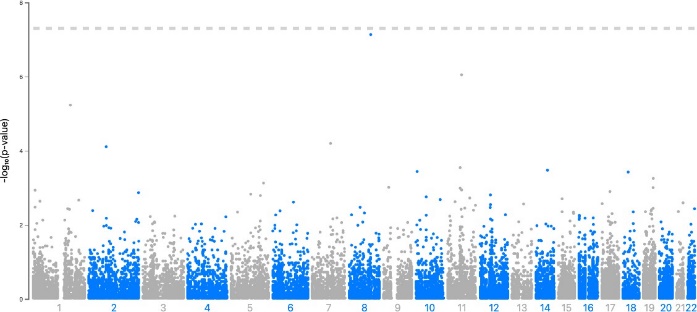

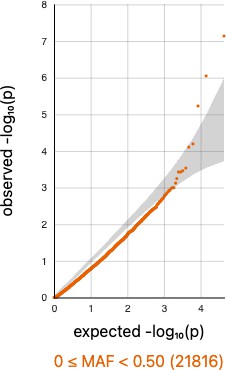


**Fig AO.** **Manhattan and quantile-quantile plots of the African ancestry meta-analysis results from METAL for any cleft (ANY).** Plots were created with LocusZoom [2]. Genomic control factor based on the median, λ_GC_, was 0.986.


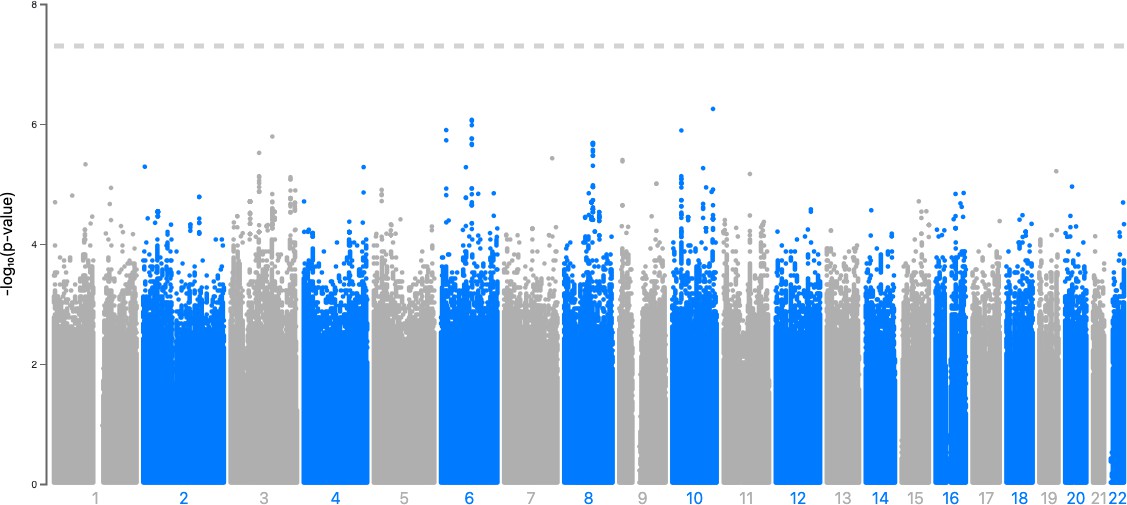

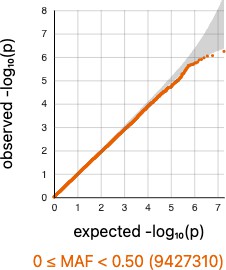


**Fig AP. Manhattan and quantile-quantile plots of the African ancestry meta-analysis results from METAL for cleft lip with/without cleft palate (CL/P).** Plots were created with LocusZoom [2]. Genomic control factor based on the median, λ_GC_, was 0.987.


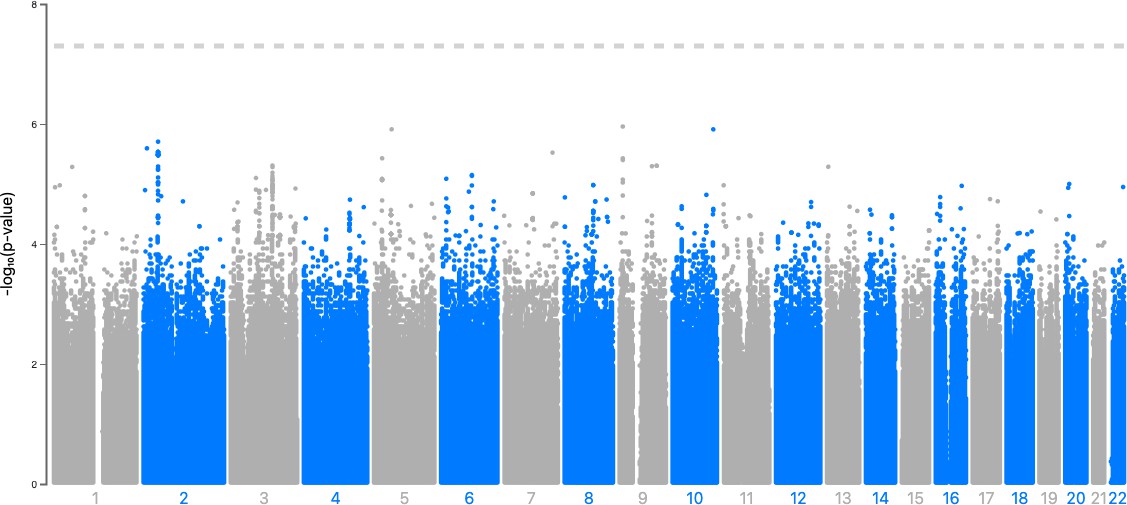

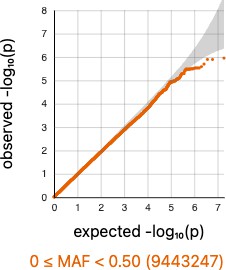


**Fig AQ.** **Manhattan and quantile-quantile plots of the African ancestry meta-analysis results from METAL for cleft lip and palate (CLP).** Plots were created with LocusZoom [2]. Genomic control factor based on the median, λ_GC_, was 0.956.


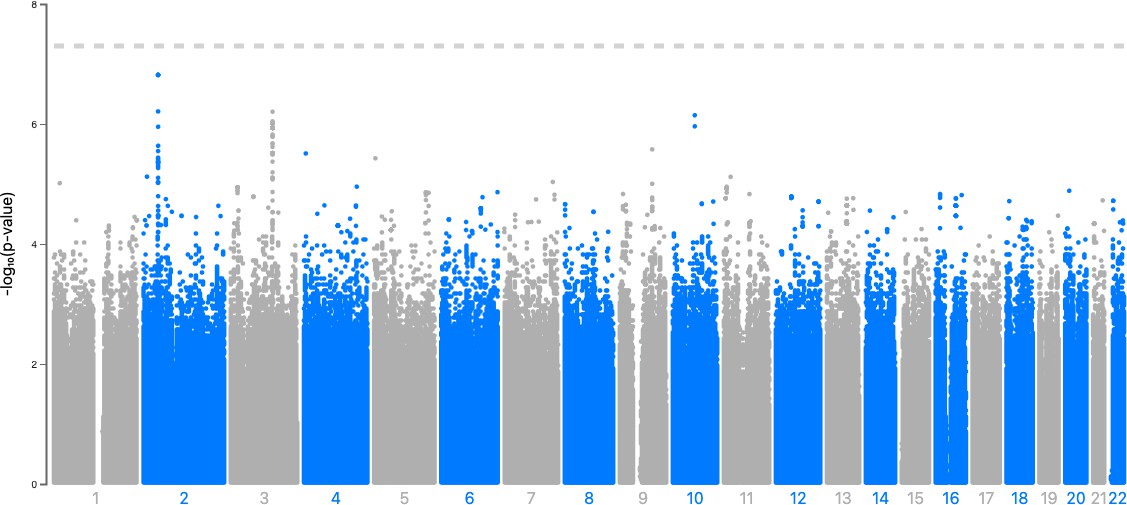

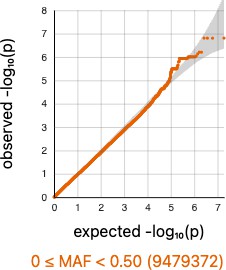


**Fig AR.** **Manhattan and quantile-quantile plots of the African ancestry meta-analysis results from METAL for cleft lip only (CLO).** Plots were created with LocusZoom [2]. Genomic control factor based on the median, λ_GC_, was 0.735.


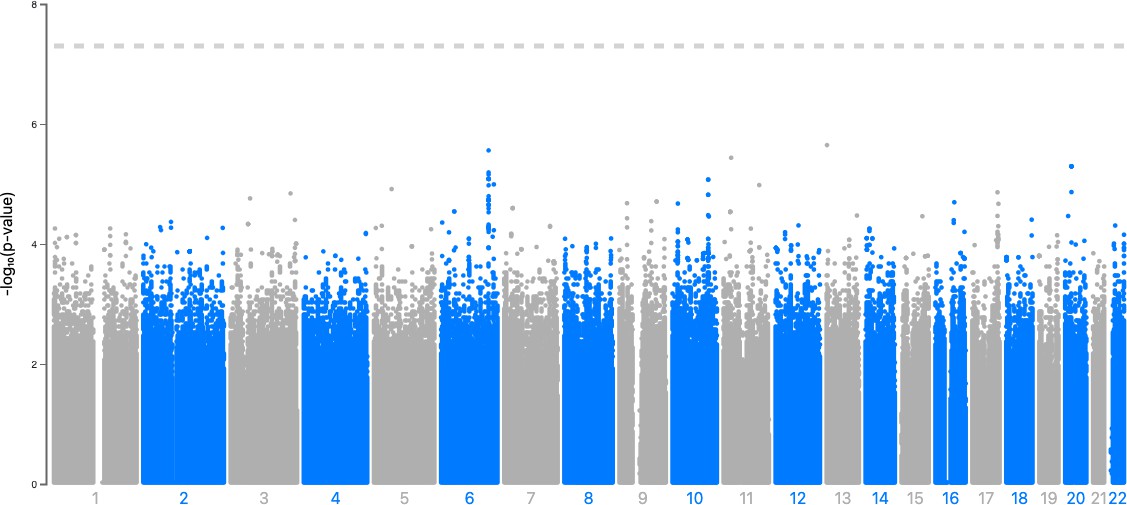

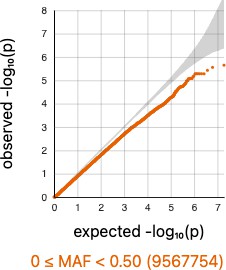


**Fig AS.** **Manhattan and quantile-quantile plots of the African ancestry meta-analysis results from METAL for cleft palate only (CPO).** Plots were created with LocusZoom [2]. Genomic control factor based on the median, λ_GC_, was 0.983.


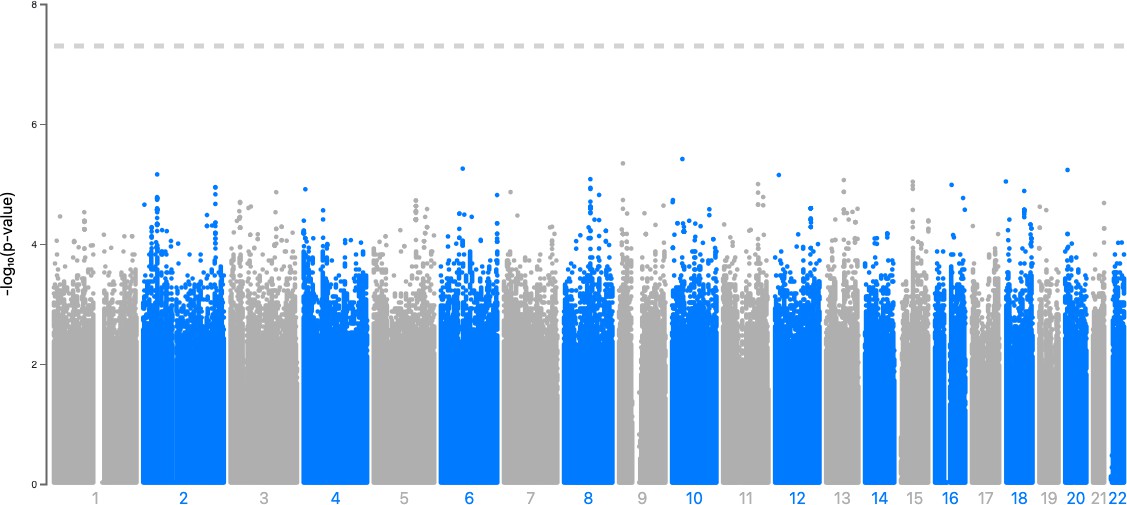

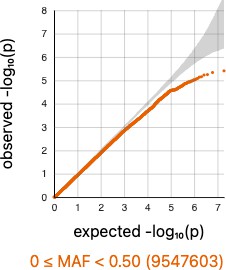


**Fig AT. Manhattan and quantile-quantile plots of the African ancestry multi-tissue TWAS results from S‑MulTiXcan for any cleft (ANY).** Plots were created with LocusZoom [2] and qqman [3]. Genomic control factor based on the median, λ_GC_, was 0.984.


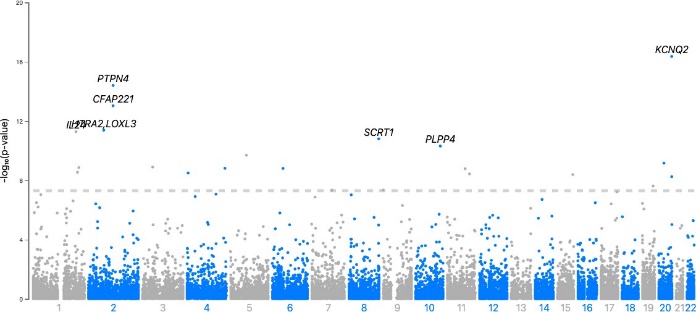

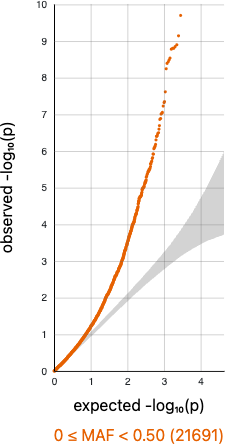


**Fig AU. Manhattan and quantile-quantile plots of the African ancestry multi-tissue TWAS results from S‑MulTiXcan cleft lip with/without cleft palate (CL/P).** Plots were created with LocusZoom [2] and qqman [3]. Genomic control factor based on the median, λ_GC_, was 0.978.


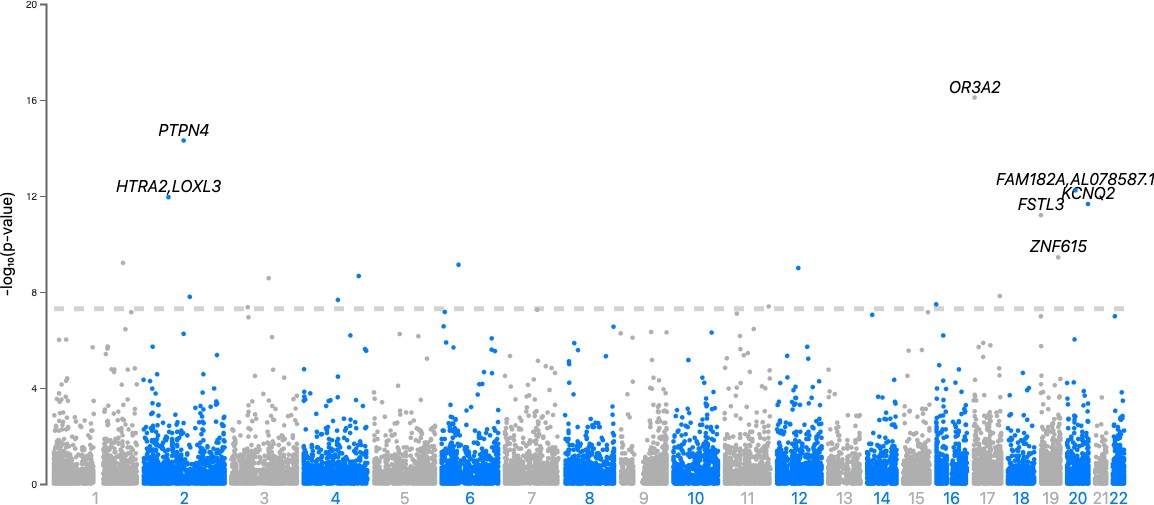

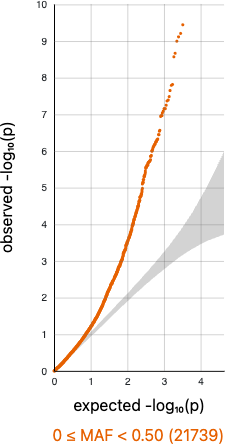


**Fig AV. Manhattan and quantile-quantile plots of the African ancestry multi-tissue TWAS results from S‑MulTiXcan for cleft lip and palate (CLP).** Plots were created with LocusZoom [2] and qqman [3]. Genomic control factor based on the median, λ_GC_, was 0.911.


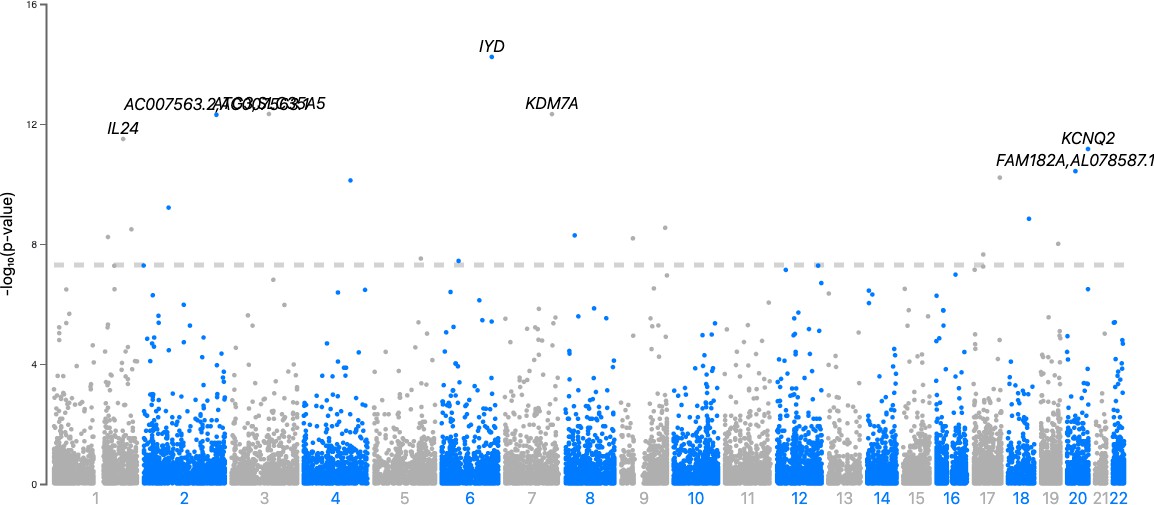

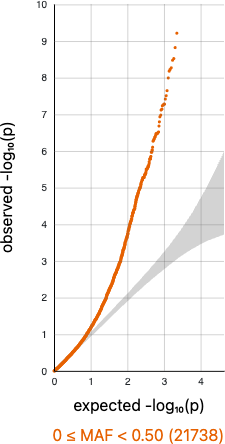


**Fig AW. Manhattan and quantile-quantile plots of the African ancestry multi-tissue TWAS results from S‑MulTiXcan for cleft lip only (CLO).** Plots were created with LocusZoom [2] and qqman [3]. Genomic control factor based on the median, λ_GC_, was 0.399.


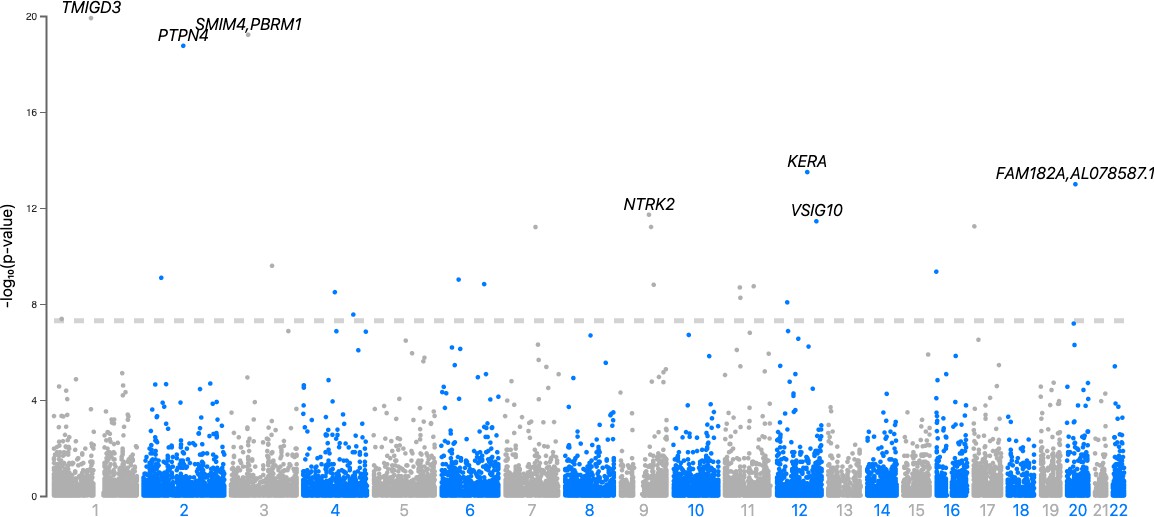

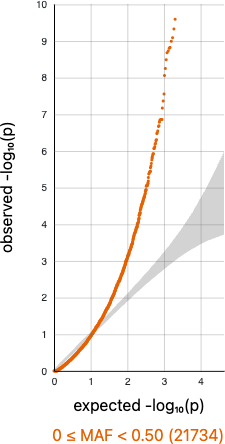


**Fig AX. Manhattan and quantile-quantile plots of the African ancestry multi-tissue TWAS results from S‑MulTiXcan for cleft palate only (CPO).** Plots were created with LocusZoom [2] and qqman [3]. Genomic control factor based on the median, λ_GC_, was 0.517.


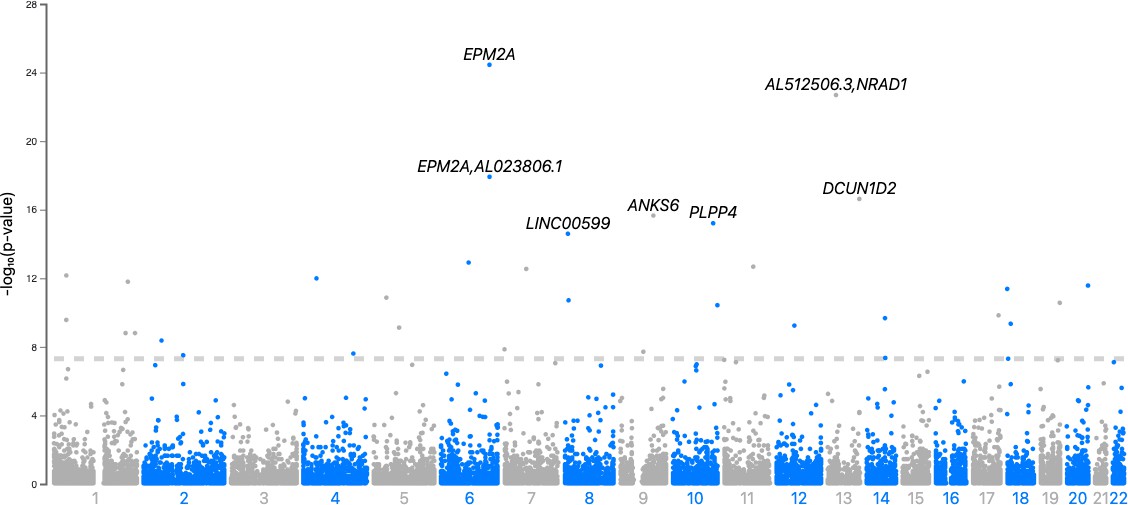

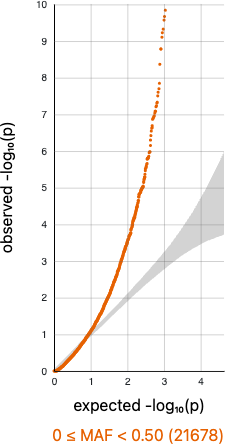


**Fig AY. Principal components of ancestry for POFC2 cohort.** Pairwise scatterplots of the first 3 principal components of ancestry are shown, with each point colored by the clustering assignment from K-means. Labels were assigned to each of these clusters based on recruitment site and self-reported race (1 = majority African ancestry, 2 = majority European ancestry, 3 = majority Central/South American ancestry, and 4 = majority Asian ancestry) to facilitate ancestry-specific meta-analyses.

**References**

1. Boughton AP, Welch RP, Flickinger M, VandeHaar P, Taliun D, Abecasis GR, et al. LocusZoom.js: interactive and embeddable visualization of genetic association study results. Bioinformatics. 2021;37: 3017–3018. doi:10.1093/bioinformatics/btab186

2. Boughton AP, Welch RP, Flickinger M, VandeHaar P, Taliun D, Abecasis GR, et al. LocusZoom.js: Interactive and embeddable visualization of genetic association study results. Bioinformatics. 2021; btab186. doi:10.1093/bioinformatics/btab186

3. Turner SD. qqman: an R package for visualizing GWAS results using Q-Q and manhattan plots. Journal of Open Source Software. 2018;3: 731. doi:10.21105/joss.00731
